# Transgenic overexpression of GTP cyclohydrolase 1 in cardiomyocytes ameliorates post-infarction cardiac remodeling

**DOI:** 10.1038/s41598-017-03234-6

**Published:** 2017-06-08

**Authors:** Yanan Liu, Shelley L. Baumgardt, Juan Fang, Yang Shi, Shigang Qiao, Zeljko J. Bosnjak, Jeannette Vásquez-Vivar, Zhengyuan Xia, David C. Warltier, Judy R. Kersten, Zhi-Dong Ge

**Affiliations:** 1 Departments of Anesthesiology, Medical College of Wisconsin, Milwaukee, 8701 Watertown Plank Road, Milwaukee, Wisconsin 53226 USA; 2 Department of Pediatrics, Medical College of Wisconsin, Milwaukee, 8701 Watertown Plank Road, Milwaukee, Wisconsin 53226 USA; 3 Department of Physiology, Medical College of Wiscosin, Milwaukee, 8701 Watertown Plank Road, Milwaukee, Wisconsin 53226 USA; 4 Department of Biophysics, Medical College of Wisconsin, Milwaukee, 8701 Watertown Plank Road, Milwaukee, Wisconsin 53226 USA; 5 Aurora Research Institute, Aurora Health Care, 750 W. Virginia Street, Milwaukee, Wisconsin 53234 USA; 6 Department of Anesthesiology, University of Hong Kong, Hong Kong, People’s Republic of China; 7 Department of Medicine, Columbia University, 630 W. 168th Street, New York, New York 10032 USA

## Abstract

GTP cyclohydrolase 1 (GCH1) and its product tetrahydrobiopterin play crucial roles in cardiovascular health and disease, yet the exact regulation and role of GCH1 in adverse cardiac remodeling after myocardial infarction are still enigmatic. Here we report that cardiac GCH1 is degraded in remodeled hearts after myocardial infarction, concomitant with increases in the thickness of interventricular septum, interstitial fibrosis, and phosphorylated p38 mitogen-activated protein kinase and decreases in left ventricular anterior wall thickness, cardiac contractility, tetrahydrobiopterin, the dimers of nitric oxide synthase, sarcoplasmic reticulum Ca^2+^ release, and the expression of sarcoplasmic reticulum Ca^2+^ handling proteins. Intriguingly, transgenic overexpression of GCH1 in cardiomyocytes reduces the thickness of interventricular septum and interstitial fibrosis and increases anterior wall thickness and cardiac contractility after infarction. Moreover, we show that GCH1 overexpression decreases phosphorylated p38 mitogen-activated protein kinase and elevates tetrahydrobiopterin levels, the dimerization and phosphorylation of neuronal nitric oxide synthase, sarcoplasmic reticulum Ca^2+^ release, and sarcoplasmic reticulum Ca^2+^ handling proteins in post-infarction remodeled hearts. Our results indicate that the pivotal role of GCH1 overexpression in post-infarction cardiac remodeling is attributable to preservation of neuronal nitric oxide synthase and sarcoplasmic reticulum Ca^2+^ handling proteins, and identify a new therapeutic target for cardiac remodeling after infarction.

## Introduction

Myocardial infarction (MI) is one of the main health and economic problems and the leading cause of death in the Western world^1, 2^. Following MI, cardiac remodeling occurs progressively in untreated patients with large MI^3^. Post-infarction cardiac remodeling is now recognized as central in the pathophysiology of advancing heart failure^3–5^. Slowing or reversing post-infarction cardiac remodeling is emerging as a therapeutic strategy for preventing the development of heart failure after MI^5, 6^. The results of conventional therapy directed at the reduction of cardiac remodeling after MI are disappointing^7, 8^. New approaches, such as stem/progenitor cell-based cardiac regeneration therapies, are shown to attenuate cardiac remodeling and/or dysfunction after MI in small animal models^9, 10^. However, in humans the therapeutic effects of stem/progenitor cell transplantation on post-infarction myocardium remain uncertain^11^. The search for new therapeutic targets to slow or reverse cardiac remodeling after MI is of importance for the precision treatment of cardiac remodeling.

GTP cyclohydrolase 1 (GCH1) is known as the first and rate-limiting enzyme in *de novo* biosynthesis of tetrahydrobiopterin (BH_4_), an essential co-factor for nitric oxide synthase (NOS), tryptophan hydroxylase, phenylalanine hydroxylase, and tyrosine hydroxylase^12^. Recent studies find that GCH1 and BH_4_ play important roles in the regulation of the expression and function of NOS, the function of the sarcoplasmic reticulum (SR) and mitochondria, oxidative/nitrosative stress, and myocyte contractility and relaxation in normal myocardium^13–15^. However, GCH1 proteins are decreased due to increased degradation of GCH1 by 26S proteasome or/and decreased biosynthesis during cardiovascular disease and in diabetes, Parkinson’s disease, or aging^16–18^. In intact animals, the deficiency or inhibition of GCH1 proteins or GCH1 gene mutations result in vascular endothelial dysfunction, systemic hypertension, pulmonary hypertension, cardiac dysfunction, or short lifespan^13, 16, 19–25^. At cellular and molecular level, insufficient GCH1 proteins lead to dysregulation of biopterins, NOS, and p38 mitogen-activated protein kinase (p38 MAPK), suppression of the SR and mitochondrial function, and oxidative/nitrosative stress^13, 14, 16, 21–23, 26^. Previous studies report that the activity of 26S proteasome, a ubiquitous enzyme responsible for the degradation of GCH1^16^, is elevated in post-infarction myocardium^27^. It is known that dysregulation of NOS isoforms, phosphorylated p38 mitogen-activated protein kinase (p-p38 MAPK), abnormalities in the SR and mitochondrial function, and oxidative/nitrosative stress contribute to the development of cardiac remodeling and heart failure^28–31^. However, how GCH1 is involved in cardiac remodeling and function after MI remains unclear.

In the present study, we examined the regulation and role of GCH1 in left ventricular remodeling after MI in mice and explored the molecular mechanisms underlying regulation of post-infarction cardiac remodeling by GCH1. First, cardiac GCH1 mRNA and proteins were kinetically measured in C57BL/6 wild-type (WT) mice from 1 to 12 weeks after MI. Secondly, the geometry and function of the left ventricle (LV), infarct size, and interstitial fibrosis were compared in the transgenic (Tg) mice with cardiomyocyte-specific overexpression of human GCH1 gene with those in C57BL/6 mice 4 weeks after MI or sham surgery^32, 33^. Lastly, to study the signaling pathways linking GCH1 with cardiac remodeling, we determined cardiac BH_4_ concentrations, free Ca^2+^ and SR Ca^2+^ release in cardiomyocytes in the presence and absence of isoproterenol, and the expression of microRNA-21, p38 MAPK, 3 isoforms of NOS, and SR Ca^2+^ handling proteins in Tg and C57BL/6 mice 4 weeks after MI or sham surgery. Our results indicate that GCH1 degradation contributes to the pathogenesis of cardiac remodeling and dysfunction after MI. More importantly, cardiomyocyte-specific overexpression of human GCH1 gene favorably regulates BH_4_, the dimerization and phosphorylation of neuronal NOS (nNOS), free Ca^2+^ and SR Ca^2+^ release in cardiomyocytes, and the expression of SR Ca^2+^ handling proteins, thereby preventing the development of cardiac remodeling after MI.

## Results

### Cardiac GCH1 is decreased in WT mice after MI

**Figure 1:**
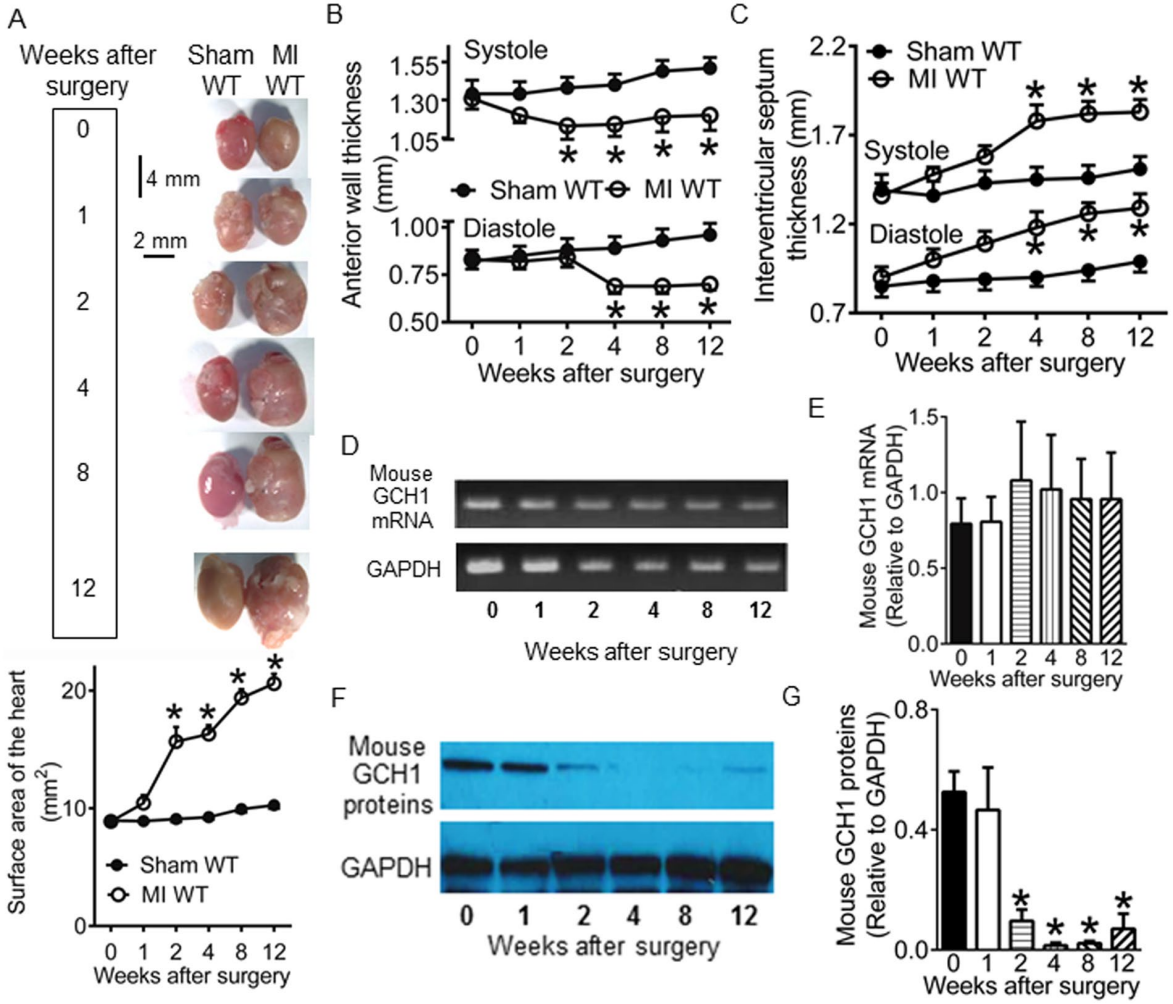
Wild-type (WT) mice developed cardiac remodeling and lost GTP cyclohydrolase 1 (GCH1) proteins following myocardial infarction (MI). (**A**) Time-dependent changes in mouse hearts after MI or sham surgery (n = 6–8 mice/group). Top: representative images of mouse hearts at baseline (0), 1, 2, 4, 8, and 12 weeks after operation. The vertical scale bar indicates 4 mm, and the horizontal scale bar indicates 2 mm. Bottom: the area of the hearts after MI or sham surgery; (**B**) The anterior wall thickness of the left ventricule after MI or sham surgery (n = 8–10 mice/group); (**C**) The thickness of interventricular septum following MI or sham surgery (n = 8–10 mice/group); (**D**) Representative PCR bands showing the expression of GCH1 mRNA and the housekeeping gene, glyceraldehyde 3-phosphate dehydrogenase (GAPDH), at 0, 1, 2, 4, 8, and 12 weeks after MI; (**E**) GCH1 mRNA normalized to GAPDH (n = 6 mice/group); (**F**) Representative Western blot bands showing the expression of cardiac mouse GCH1 proteins and GAPDH proteins in WT mice following MI; (**G**) Expression of mouse GCH1 proteins normalized to GAPDH proteins (n = 6 mice/group). *P < 0.05 versus sham WT groups at corresponding time points (**A**,**B** and **C**) or 0 weeks (**G**).

We first examined whether cardiac GCH1 gene and proteins are regulated in remodeled hearts after MI in C57BL/6 mice. C57BL/6 mice underwent permanent ligation of the left coronary artery to make MI or sham surgery as control. Coronary artery ligation resulted in significant changes in heart shape and increases in heart size from 2 to 12 weeks after MI (P < 0.05 between sham WT and MI WT groups, n = 6–8 mice/group) (Fig. 1A). We used a noninvasive transthoracic echocardiography to measure the wall thickness of the LV at 0 (baseline), 1, 2, 4, 8, and 12 weeks after surgery. The thickness of left ventricular wall was comparable between sham WT and MI WT groups at baseline (P > 0.05, n = 10 mice/group) (Fig. 1B and C). Compared with sham WT groups, anterior wall was thinner at both end diastole and end systole, and interventricular septum was thicker at both end diastole and end systole from 2 to 12 weeks after surgery (P < 0.05, n = 8–10 mice/group). After echocardiographic examination was completed, the LVs were harvested for determination of GCH1 mRNA and proteins. The levels of GCH1 mRNA and proteins were comparable between MI WT and sham WT groups at baseline. The levels of GCH1 mRNA were not changed over time in both MI WT and sham WT groups 1 to 12 weeks after surgery (Fig. 1D and E). However, the expression of GCH1 proteins was significantly decreased 2 to 12 weeks after MI (P < 0.05 versus 0 week, n = 6 mice/group) (Fig. 1F and 1G). These results suggest that GCH1 proteins are degraded in post-infarction remodeled myocardium of WT mice.

### Cardiomyocyte-specific overexpression of GCH1 diminishes cardiac remodeling after MI

**Figure 2:**
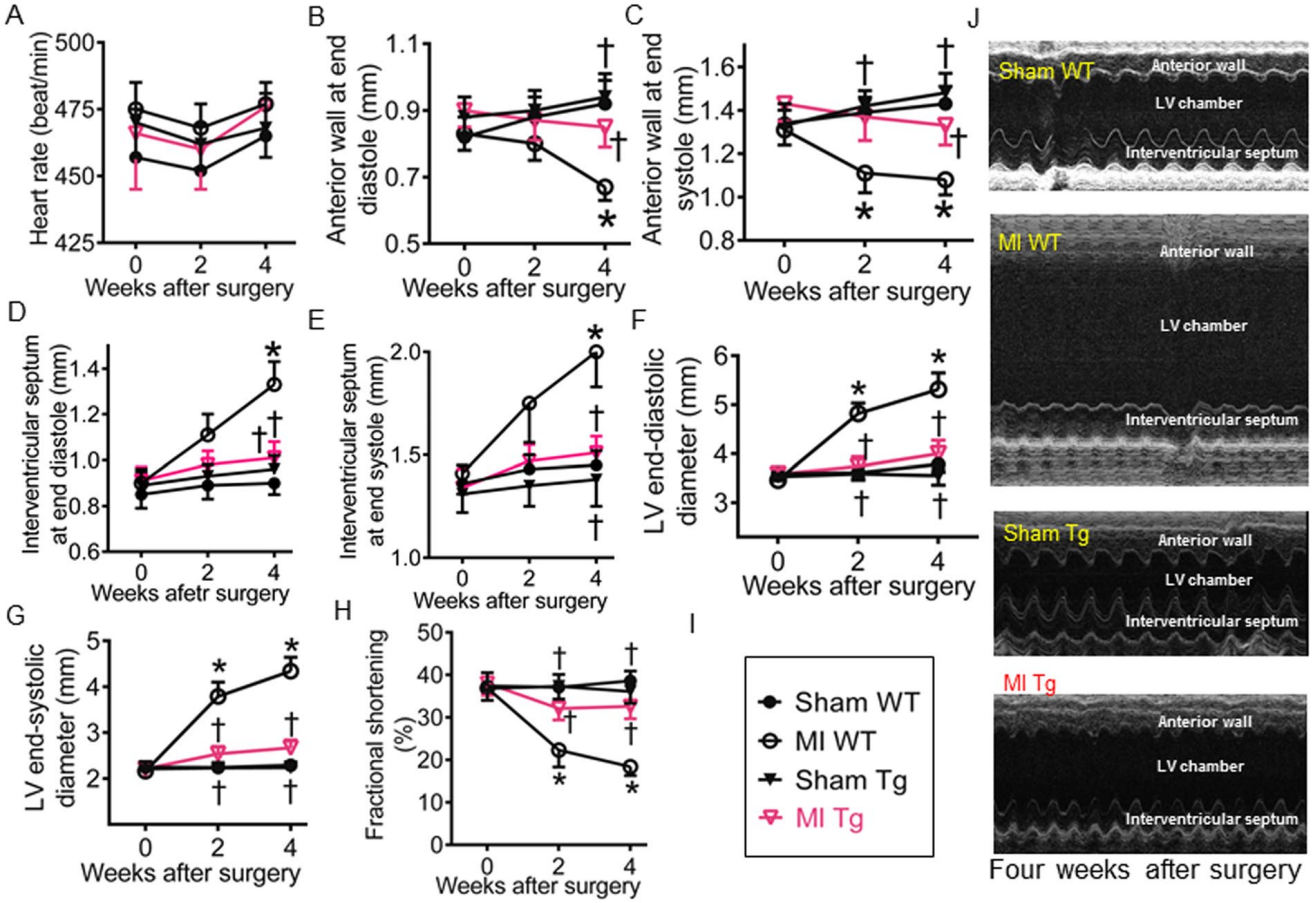
Effects of GTP cyclohydrolase 1 (GCH1) overexpression on cardiac remodeling 4 weeks after myocardial infarction (MI). (**A**) Heart rate; (**B**) The anterior wall thickness of the left ventricle (LV) at end diastole: (**C**) The anterior wall thickness of the LV at end systole; (**D**) The interventricular septum thickness of the LV at end diastole: (**E**) The interventricular septum thickness of the LV at end systole; (**F**) LV end-diastolic diameter; (**G**) LV end-systolic diameter; (**H**) Fractional shortening; (**I**) Figure legend: sham WT (wild-type), MI WT, sham Tg (GCH1 transgene), and MI Tg; (**J**) representative M-mode echocardiograms showing anterior wall, LV chamber, and interventricular septum in the mice 4 weeks after MI. *P < 0.05 versus sham WT groups; ^†^P < 0.05 versus MI WT groups (n = 8–12 mice/group).

To study whether increases in cardiac GCH1 can attenuate cardiac remodeling after MI, we made infarction in Tg and C57BL/6 WT mice by ligating the left coronary artery. Sham control animals underwent the same procedures except coronary artery ligation. The geometry of the LV was evaluated with an echocardiography at 0 (baseline), 2, and 4 weeks after surgery. Heart rate, the thickness of left ventricular anterior and posterior walls, internal diameters, and fractional shortening were comparable among 4 groups of mice at baseline (P > 0.05, n = 12 mice/group) (Fig. 2). Compared with sham WT groups, the thickness of LV anterior wall at both end diastole and end systole and fractional shortening were significantly decreased, whereas the thickness of interventricular septum at both end diastole and end systole and the internal diameters of left ventricular chamber were significantly increased in MI WT but not MI Tg groups 4 weeks after MI (P < 0.05, n = 8–12 mice/group]. There were no significant differences in the wall thickness and diameter of the LV and fractional shortening between MI Tg and sham WT groups throughout the experiments (P > 0.05). These results indicate that GCH1 overexpression attenuates cardiac remodeling after MI.

### GCH1 overexpression attenuates infarct size and interstitial fibrosis

**Figure 3:**
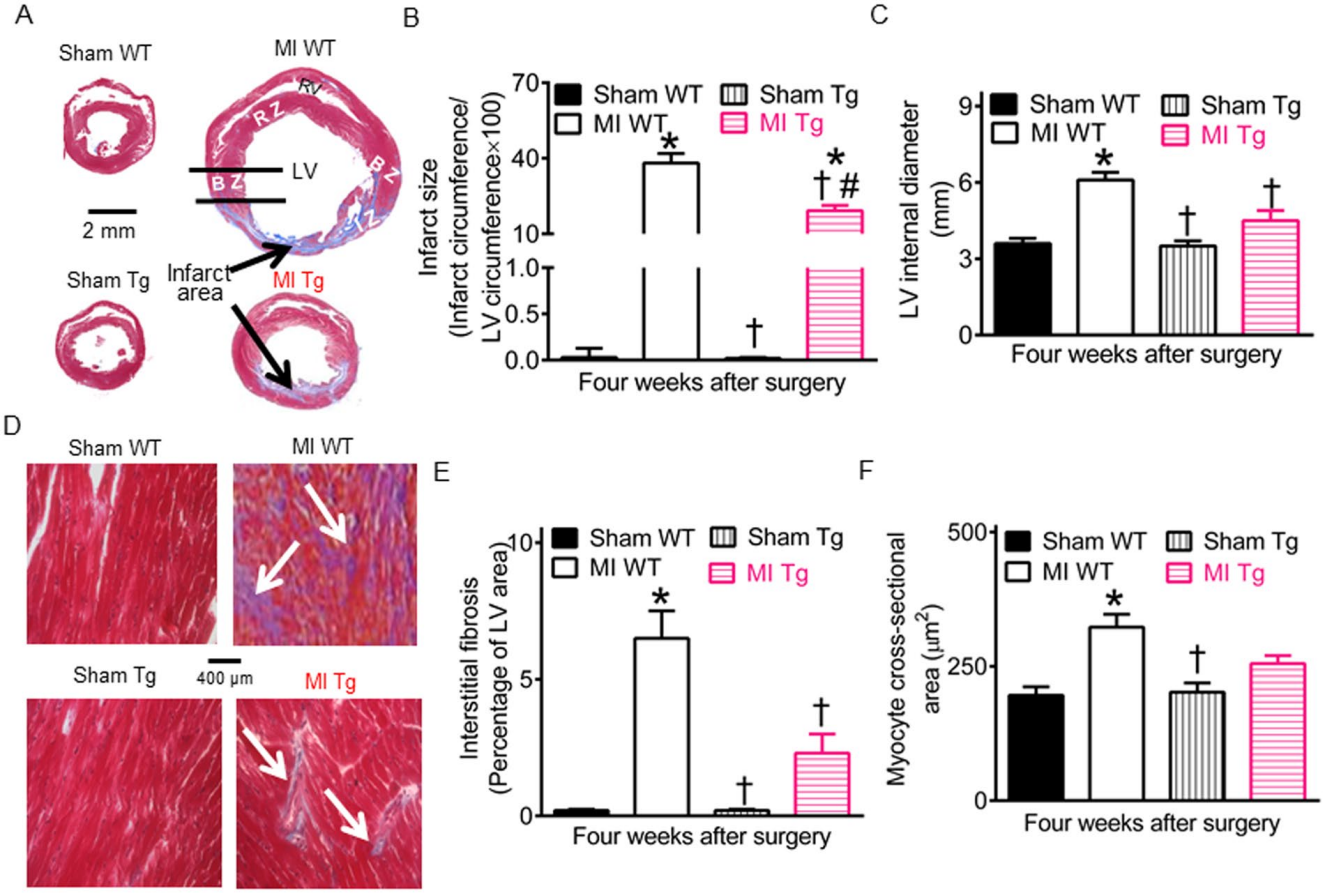
Effects of GTP cyclohydrolase 1 (GCH1) overexpression on myocardial infarct size and interstitial fibrosis after myocardial infarction (MI). (**A**) Representative Masson’s trichrome-stained cross section of the heart showing infarct area with fibrosis. The scale bar shows 2 mm, and the arrows indicate infarct area with fibrosis. BZ, border zone; IZ, infarct zone; LV, left ventricle; RV, right ventricle; RZ, remote zone; (**B**) Infarct size expressed as a percentage of infarct circumference/total circumference of the LV; (**C**) LV internal diameters at the papillary muscle levels; (**D**) Masson’s trichrome-stained myocardium showing interstitial fibrosis. The scale bar shows 400 µm, and the arrows indicate fibrosis; (**E**) Interstitial fibrosis expressed as a percentage of LV area; (**F**) Myocyte cross-sectional area. Mouse hearts were stained with Masson’s trichrome in wild-type (WT) and transgenic (Tg) mice 4 weeks after MI or sham surgery. *P < 0.05 versus sham WT groups; ^†^P < 0.05 versus MI WT groups; ^#^P < 0.05 versus sham Tg groups (n = 8–10 mice/group).

Infarct size, LV diameters, and interstitial fibrosis in remodeled hearts are associated with the development of heart failure^3, 34^. We measured infarct size, LV internal diameters, interstitial fibrosis, and myocyte cross-sectional area in Masson’s trichrome-stained sections of mouse hearts. The sections obtained from sham WT and sham Tg mice showed no infarction (Fig. 3A). In contrast, the sections obtained from MI WT mice 4 weeks after MI revealed an anteroapical infarct that extended into the anterolateral wall (infarct zone, IZ) at the level of the papillary muscle, whereas the interventricular septum (remote zone, RZ) was generally sparred (Fig. 3A). Infarct size expressed as a percentage of infarct circumference/LV circumference was 38 ± 4%, and LV internal diameter at mid-ventricular level was 6.1 ± 0.3 mm (n = 10 mice) in MI WT group (Fig. 3B and C). GCH1 overexpression significantly decreased infarct size and LV internal diameters to 19 ± 2% and 4.5 ± 0.4 mm (P < 0.05 between MI Tg and MI WT groups, n = 10 mice/group), respectively (Fig. 3B and C). In RZ of the LV, interstitial fibrosis and myocyte cross-sectional area were significantly increased in MI WT but not MI Tg groups compared with sham WT groups (Fig. 3D,E and F).

**Figure 4:**
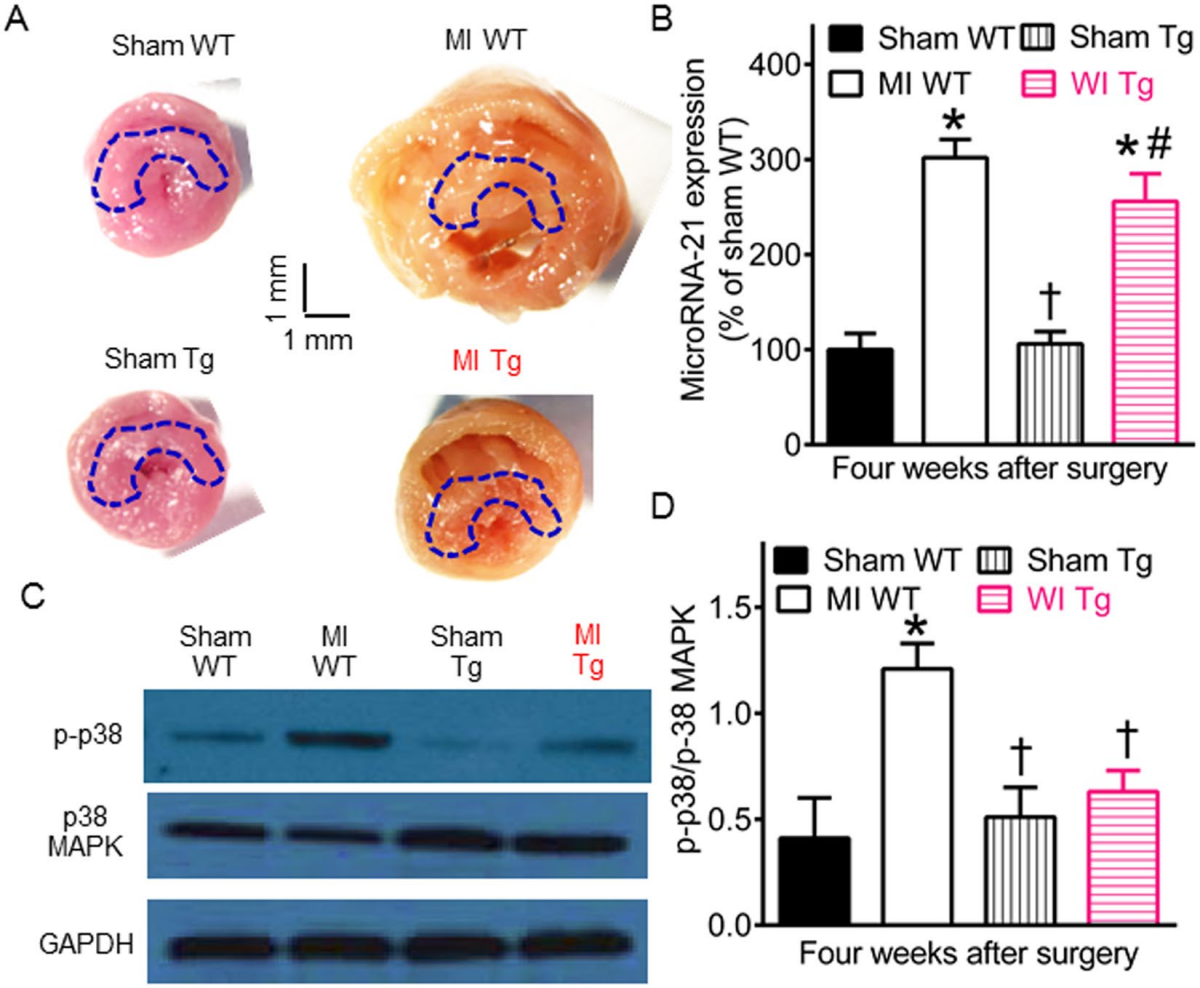
Effects of GTP cyclohydrolase 1 (GCH1) overexpression on microRNA-21 and p38 mitogen-activated protein kinase (MAPK) after myocardial infarction (MI). (**A**) Representative transverse sections of fresh mouse hearts showing the positon of tissue sampling for measurements of microRNA-21 and p38 MAPK 4 weeks after (MI) or sham surgery. The drawing areas indicate the position of tissue sampling, and the scales indicate 1 mm. (**B**) The expression of microRNA-21 mRNA; (**C**) representative Western blot bands showing the expression of phosphorylated p38 (p-p38) MAPK, total p38 MAPK, and GAPDH; (**D**) The ratio of phosphorylated p38 (p-p38) MAPK/p38 MAPK. *P < 0.05 versus sham WT groups; ^†^P < 0.05 versus MI WT groups; ^#^P < 0.05 versus sham Tg groups (n = 8–10 mice/group).

Either MicroRNA-21 or p38 MAPK is associated with the pathogenesis of interstitial fibrosis^35, 36^. To study the molecular mechanisms underlying reduced interstitial fibrosis by GCH1 overexpression, we determined the expression of microRNA-21 and p38 MAPK in the RZ of myocardium in WT and Tg mice 4 weeks after MI or sham surgery (Fig. 4A). Compared with sham WT groups, microRNA-21 levels and the ratio of p-p38 MAPK/p38 MAPK were significantly increased in MI WT groups (P < 0.05, n = 5 mice/group) (Fig. 4B,C and D). There were no significant differences in myocardial microRNA-21 levels and the ratio of p-p38/p38 MAPK between sham Tg and sham WT groups (P > 0.05). Interestingly, GCH1 overexpression significantly decreased the ratio of p-p38/p38 MAPK (P < 0.05 between MI Tg and MI WT groups) but not microRNA-21 levels after MI (Fig. 4B and D). Thus, GCH1 overexpression inhibits the formation of interstitial fibrosis through reduction of p-p38 MAPK.

### GCH1 overexpression improves cardiac function after MI

**Figure 5:**
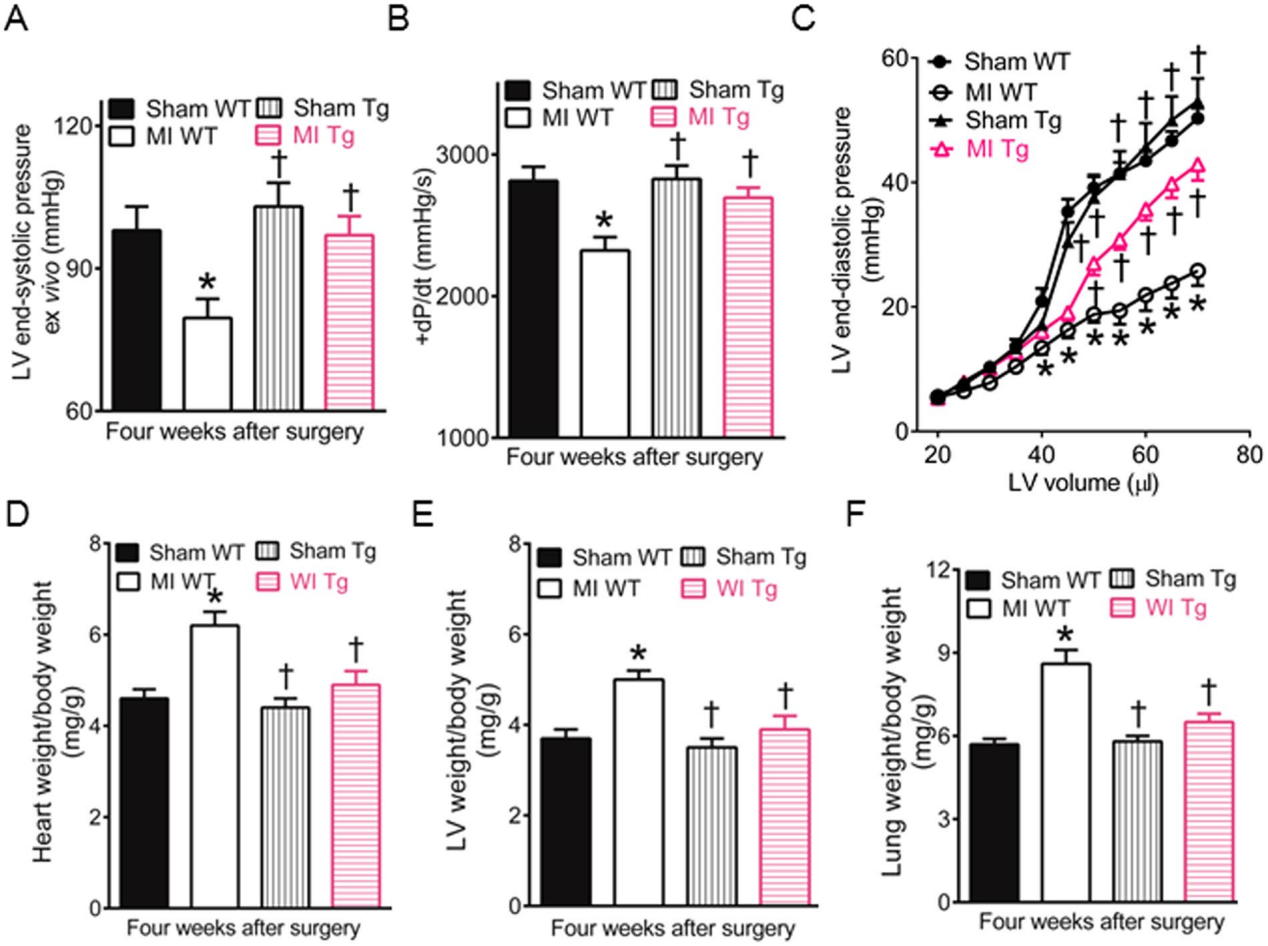
GTP cyclohydrolase 1 (GCH1) overexpression improves cardiac function 4 weeks after myocardial infarction (MI). (**A**) Left ventricular (LV) end-systolic pressure; (**B**) The rate of LV pressure rise (+dP/dt); (**C**) The LV end-diastolic pressure-volume relationship; (**D**) Heart weight normalized to body weight; (**E**) LV weight normalized to body weight: (**F**) Lung weight normalized to body weight. Figure legend: sham WT, wild-type mice receiving sham surgery; MI WT, wild-type mice undergoing myocardial infarction; sham Tg, transgenic GCH1 mice receiving sham surgery; MI Tg, transgenic GCH1 mice undergoing myocardial infarction. *P < 0.05 versus sham WT groups; ^†^P < 0.05 versus MI WT groups (n = 8–10 mice/group).

The Langendorff-perfused isolated mouse hearts offer a highly thorough and reliable model for the analysis of myocardial contractility and relaxation^37, 38^. Since many factors can influence cardiac performance *in vivo* (Fig. S1), we evaluated the effects of GCH1 overexpression on cardiac function after MI in the isovolumic, buffer-perfused, balloon-in-LV Langendorff preparation. LV end-systolic pressure and the value of + dP/dt (the rate of LV pressure rise) were significantly smaller in MI WT than sham WT groups (P < 0.05, n = 8–11 hearts/group) (Fig. 5A,B and C). Compared with MI WT groups, they were significantly elevated in MI Tg groups (P < 0.05) (Fig. 5B and C), suggesting that GCH1 overexpression elevates cardiac contractility after MI. LV end-diastolic pressure was comparable among 4 groups of mice at 20, 25, 30, and 35 µl of LV volume (P > 0.05, n = 8–11 hearts/group) (Fig. 5D). Compared with sham WT groups, LV end-diastolic pressure was significantly decreased in MI WT but not MI Tg groups from 40 to 70 µl of LV volume (Fig. 5D), suggesting that GCH1 overexpression improves cardiac relaxation after MI.

Body weight was comparable among 4 groups of mice 4 weeks after MI or sham surgery (Table S2). However, the ratios of heart weight/body weight, LV weight/body weight, and lung weight/body weight were significantly higher in MI WT than sham WT groups (P < 0.05, n = 8–10 mice/group) (Fig. 5E,F and G). These parameters were comparable between sham Tg and sham WT groups. Compared with MI WT groups, the ratios of heart weight/body weight, LV weight/body weight, and lung weight/body weight were significantly decreased in MI Tg groups (Table S2), suggesting that GCH1 overexpression reduces LV mass after MI and lung edema arising from left ventricular dysfunction.

### GCH1 overexpression reduces cardiomyocyte Ca^2+^ overload after MI

**Figure 6:**
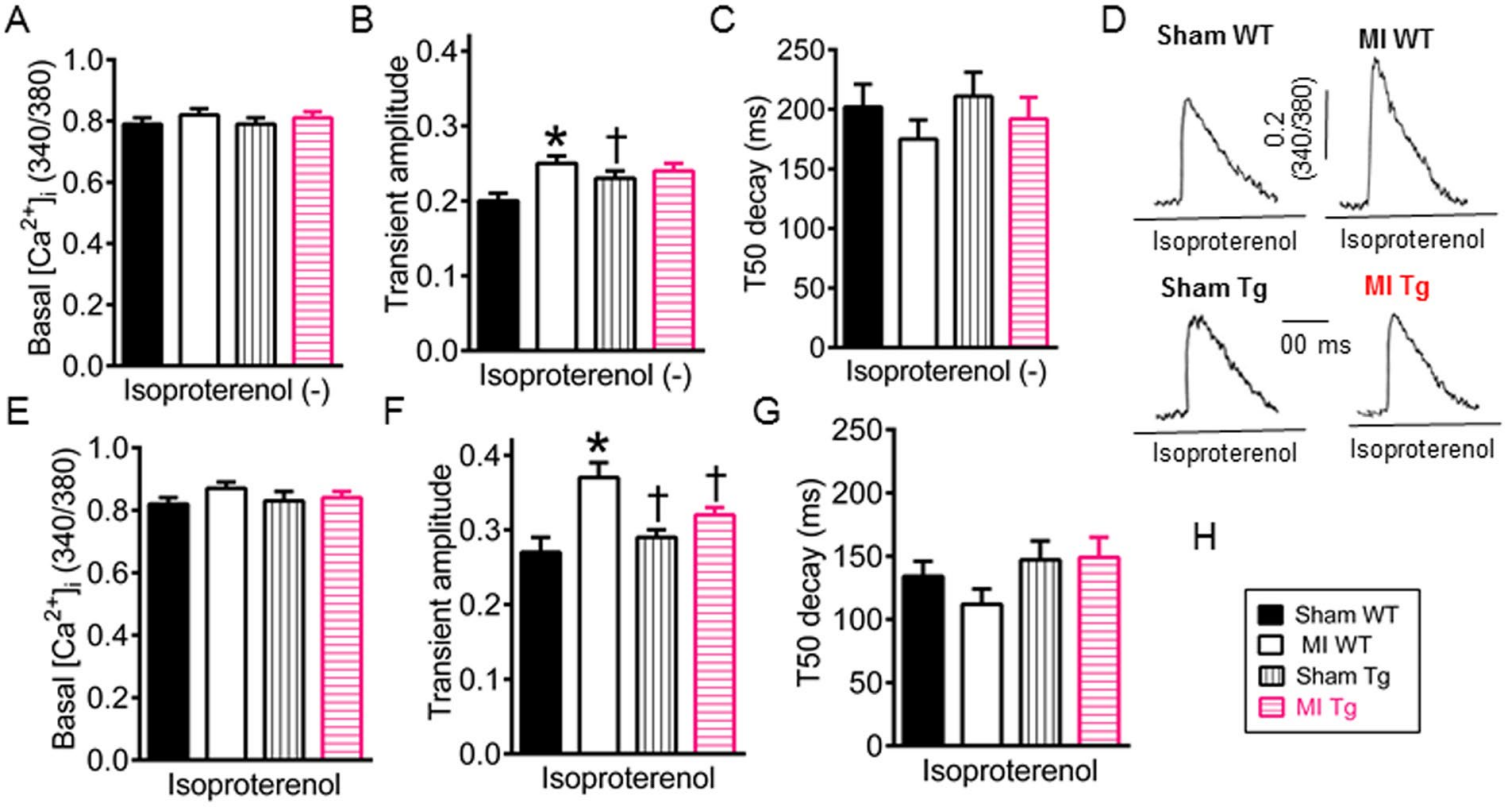
Effects of GTP cyclohydrolase 1 (GCH1) overexpression on intracellular [Ca^2+^]_i_ after myocardial infarction (MI). (**A**) Basal Ca^2+^ concentrations in the absence of isoproterenol; (**B**) The amplitude of Ca^2+^ transients elicited by electrical stimulation in the absence of isoproterenol; (**C**) Time to 50% decay (T50 decay) of Ca^2+^ transients in the absence of isoproterenol. (**D**) Original recordings of Ca^2+^ transients stimulated by electricity in the presence of isoproterenol in cardiomyocytes isolated from transgenic (Tg) and wild-type (WT) mice 4 weeks after MI or sham surgery. The vertical scale bar indicates 0.2 Fura-2 ratio (340/380 nM) unit, and the horizontal scale bar indicates 100 ms; (**E**) Basal [Ca^2+^]_i_ in the presence of isoproterenol; (**F**) Ca^2+^ transient amplitude in the presence of isoproterenol; (**G**) T50 decay of Ca^2+^ transients in the presence of isoproterenol; (**H**) Figure legend. *P < 0.05 versus sham WT groups; ^†^P < 0.05 versus MI WT groups (n = 63–66 cells/goup).

Intracellular free Ca^2+^ plays a central part in regulating excitation-contraction coupling of cardiac muscle and in modulating systolic and diastolic function in the heart^39^. Perturbations in intracellular Ca^2+^ handling contribute to contractile dysfunction in various models of cardiac dysfunction^40, 41^. We measured intracellular free Ca^2+^ in Fura-2-loaded cardiomyocytes isolated from WT and Tg mice 4 weeks after MI or sham surgery (Fig. 6). There were no significant differences in basal Ca^2+^ concentrations and time to 50% decay (T50) of Ca^2+^ transients elicited by electric field stimulation among 4 groups of mice (P > 0.05, n = 63–66 cells/group) (Fig. 6A and C). Compared with sham WT group, Ca^2+^ transient amplitude was greater in MI WT but not MI Tg groups (P < 0.05) (Fig. 6B).

In intact animals, cardiomyocytes live in the presence of β-adrenergic agonists such as epinephrine, norepinephrine, and dopamine^42^. We further determined intracellular [Ca^2+^]_I_ in the presence of the β-adrenergic agonist, isoproterenol (Fig. 6D). Application of 20 nM isoproterenol to cardiomyocytes elevated basal Ca^2+^ levels and Ca^2+^ transient amplitude and decreased T50 decay of Ca^2+^ transients elicited by electricity in cardiomyocytes isolated from all mice. In the presence of isoproterenol, basal [Ca^2+^]_I_ and T50 decay of Ca^2+^ transients were comparable among 4 groups of mice (P > 0.05) (Fig. 6E and G). Compared with sham WT group, Ca^2+^ transient amplitude was significantly increased in MI WT but not in MI Tg group (P < 0.05, n = 63–66 cells/group) (Fig. 6F). There were no significant differences in Ca^2+^ transient amplitude between MI Tg and sham WT groups (P > 0.05) (Fig. 6F). These results suggest that MI WT cardiomyocytes have excessive cytoplasm Ca^2+^ (Ca^2+^ overload), and GCH1 overexpression reduces Ca^2+^ overload after MI.

### GCH1 overexpression preserves SR Ca^2+^ release and SR Ca^2+^ handling proteins after MI

**Figure 7:**
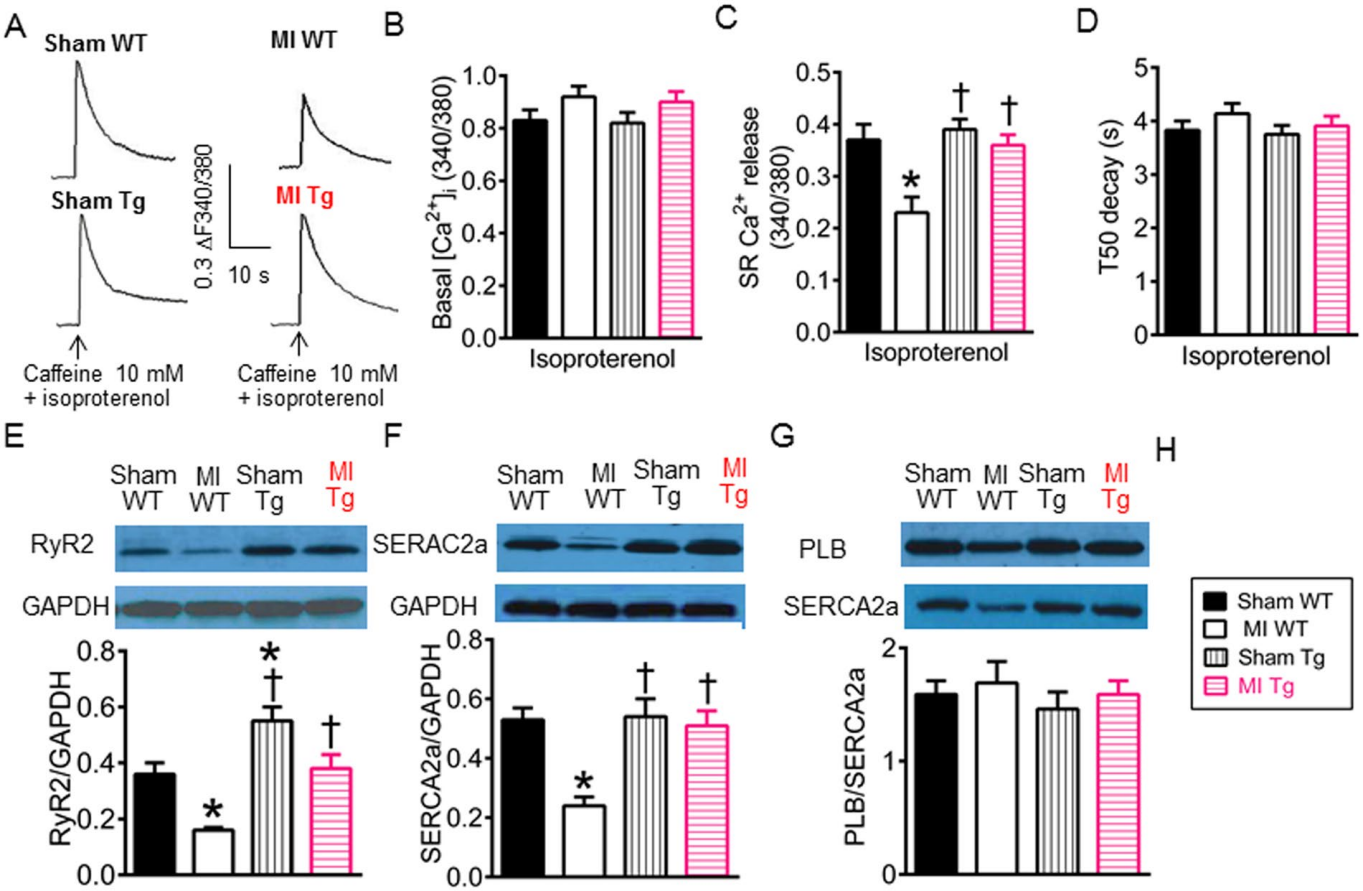
Effects of GTP cyclohydrolase 1 (GCH1) overexpression on sarcoplasmic reticulum (SR) Ca^2+^ release and Ca^2+^ handling proteins after myocardial infarction (MI). (**A**) Original recordings of caffeine-induced Ca^2+^ release in cardiomyocytes isolated from wild-type (WT) and transgenic (Tg) mice 4 weeks after MI or sham surgery in 0 Na^+^ and 0 Ca^2+^ Tyrode buffer under isoproterenol stimulation. The vertical scale bar indicates 0.3 Fura-2 ratio unit, and the horizontal bar indicates 10 s; (**B**) Basal [Ca^2+^]_I_ in the presence of isoproterenol; (**C**) SR Ca^2+^ release in the presence of isoproterenol (n = 63–66 cells/group); (**D**) Time to 50% decay (T50 decay) of caffeine-induced Ca^2+^ transients in the presence of isoproterenol; (**E**) The expression of ryanodine receptors (RyR2) normalized to glyceraldehyde 3-phosphate dehydrogenase (GAPDH) in Tg and WT mice 4 weeks after MI or sham surgery (n = 5 mice/group). Top: Wesern blot bands showing the expression of RyRs and GAPDH. Bottom: the ratio of RyRs/GAPDH; (**F**) The expression of SR Ca^2+^-ATPase (SERCA2a) proteins normalized to GAPDH. Top: Western blot bands showing the expression of SERCA2a and GAPDH. Bottom: the ratio of SERCA2a/GAPDH; (**G**) The expression of phospholamban (PLB) and SERCA2a in Tg and WT mice 4 weeks after MI or sham surgery. Top: Western blot bands showing the expression of PLB and SERCA2a. Bottom: the ratio of PLB/SERCA2a. *P < 0.05 versus sham WT groups; ^†^P < 0.05 versus MI WT groups.

The SR plays critical roles in intracellular Ca^2+^ release and re-uptake^43^. To study whether Ca^2+^ overload also exists in the SR, we quantified SR Ca^2+^ release by applying 10 mM caffeine to cardiomyocytes in 0 Na^+^ and 0 Ca^2+^ Tyrode buffer^16^ (Fig. 7A). In the presence of 20 nM isoproterenol, SR Ca^2+^ release was significantly lower in MI WT group (P < 0.05, n = 63–66 cells/group) but not in MI Tg group than sham WT group (Fig. 7C). There were no significant differences in SR Ca^2+^ release between MI Tg and sham WT groups (P > 0.05). These results indicate that SR Ca^2+^ release is impaired in MI WT cardiomyocytes, and GCH1 overexpression preserves SR Ca^2+^ release after MI.

In cardiomyocytes, ryanodine receptors (RyR2) control Ca^2+^ release from the SR to trigger muscle contraction, whereas SR Ca^2+^-ATPase (SERCA2a) re-uptakes Ca^2+^ from the cytosol into the SR to elicit relaxation^43, 44^. We used Western blot analysis to measure the expression of SR Ca^2+^ handling proteins in WT and Tg mouse hearts 4 weeks after MI or sham surgery. Compared with sham WT groups, the ratios of RyR2/glyceraldehyde 3-phosphate dehydrogenase (GAPDH) and SERCA2a/GAPDH were significant decreased in MI WT (P < 0.05, n = 5 mice/group) but not MI Tg groups (Fig. 7E and F). SERCA2a activity is directly regulated by the naturally inhibitory phospholamban (PLB)^45^. Furthermore, we examined the effects of GCH1 on the expression of PLB and SERCA2a. Compared with sham WT group, the ratio of PLB/SERCA2a was not significant altered in either MI WT or MI Tg groups (P > 0.05) (Fig. 7G), suggesting that the activity of SERCA2a is not changed in post-infarction remodeled myocardium. Thus, decreases in RyR2 and SERCA2a contribute to impaired SR Ca^2+^ release in MI WT mice, and preservation of SR Ca^2+^ handling proteins by GCH1 overexpression is responsible for improvement in the SR Ca^2+^ release after MI.

### GCH1 overexpression elevates cardiac BH_4_ levels after MI

**Figure 8:**
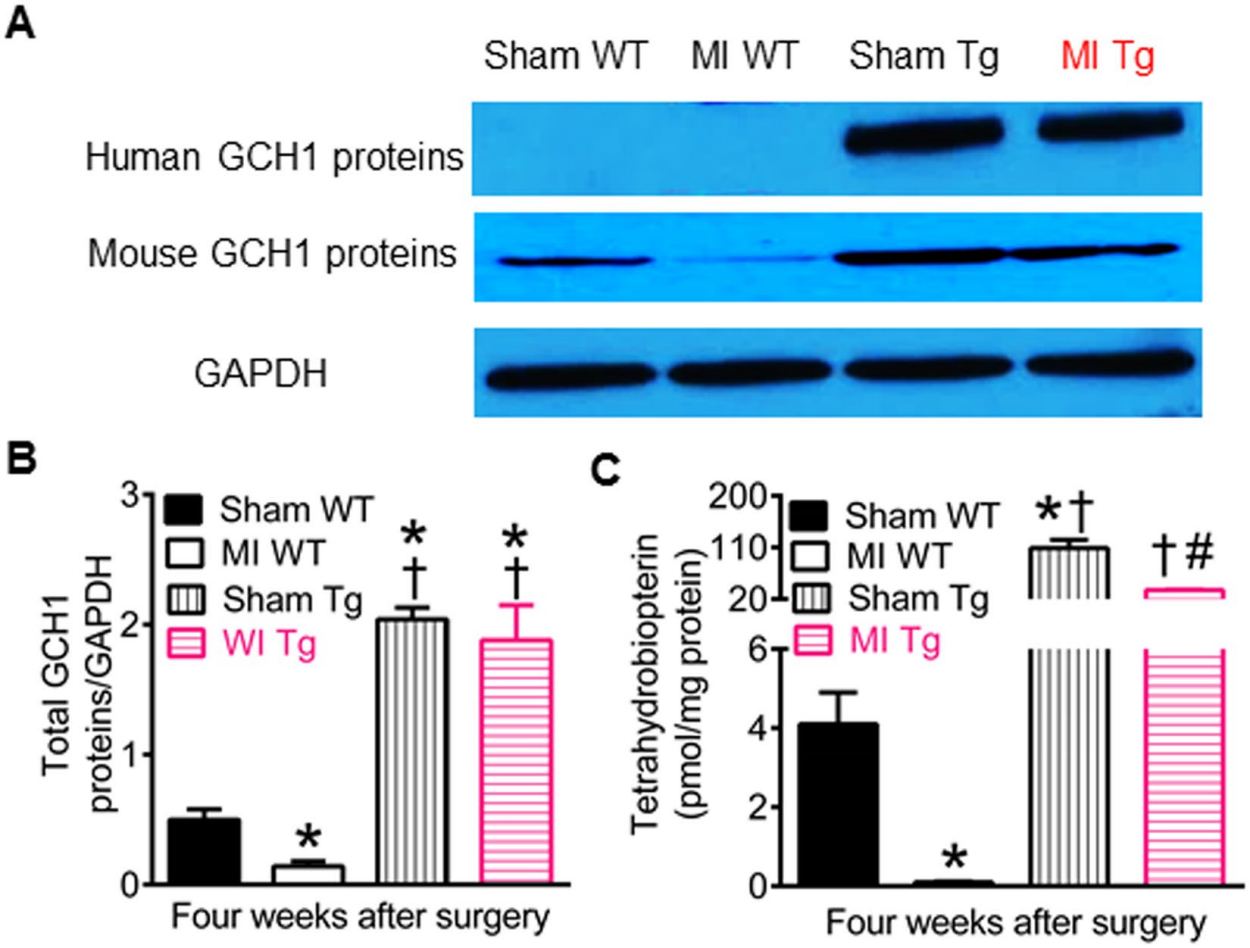
Effects of transgenic overexpression of human GTP cyclohydrolase 1 (GCH1) on GCH1 proteins and tetrahydrobiopterin in post-infarction remodeled myocardium. (**A**) Representative Western blot bands showing the expression of human and mouse GCH1 proteins and glyceraldehyde 3-phosphate dehydrogenase (GAPDH) as control in wild-type (WT) and transgenic (Tg) mice 4 weeks after myocardial infarction (MI) or sham surgery; (**B**) Total (human + mouse) GCH1 proteins normalized to GAPDH; C: cardiac tetrahydrobiopterin concentrations. *P < 0.05 versus sham WT groups; ^†^P < 0.05 versus MI WT groups; ^#^P < 0.05 versus sham Tg groups (n = 5–6 mice/group).

To examine whether transfer of human GCH1 gene effectively elevates the expression of GCH1 proteins in Tg mice, we used Western blot analysis to determine the expression of human and mouse GCH1 proteins in post-infarction remodeled hearts. Transfer of human GCH1 gene drove GCH1 protein expression in Tg mouse hearts with and without cardiac remodeling (Fig. 8A). Compared with sham WT group, the expression of mouse GCH1 proteins was significantly decreased in MI WT group 4 weeks after MI, however, the expression of total (human plus mouse) GCH1 proteins was significantly elevated in both sham Tg and MI Tg groups (P < 0.05, n = 5–6 mice/group) (Fig. 8A and B).

BH_4_ plays important roles in cardiovascular health and disease^12, 46^. GCH1 is the rate-limiting enzyme in *de novo* synthesis of BH_4_^12^. We quantified cardiac BH_4_ concentrations with high-performance liquid chromatography (HPLC)^38^. Compared with sham WT groups, myocardial BH_4_ concentrations were significantly decreased in MI WT groups and increased in sham Tg and MI Tg groups (P < 0.05, n = 6 mice/group) (Fig. 8C), which was similar to changes in GCH1.

### GCH1 overexpression elevates nNOS dimerization and phosphorylation after MI

**Figure 9:**
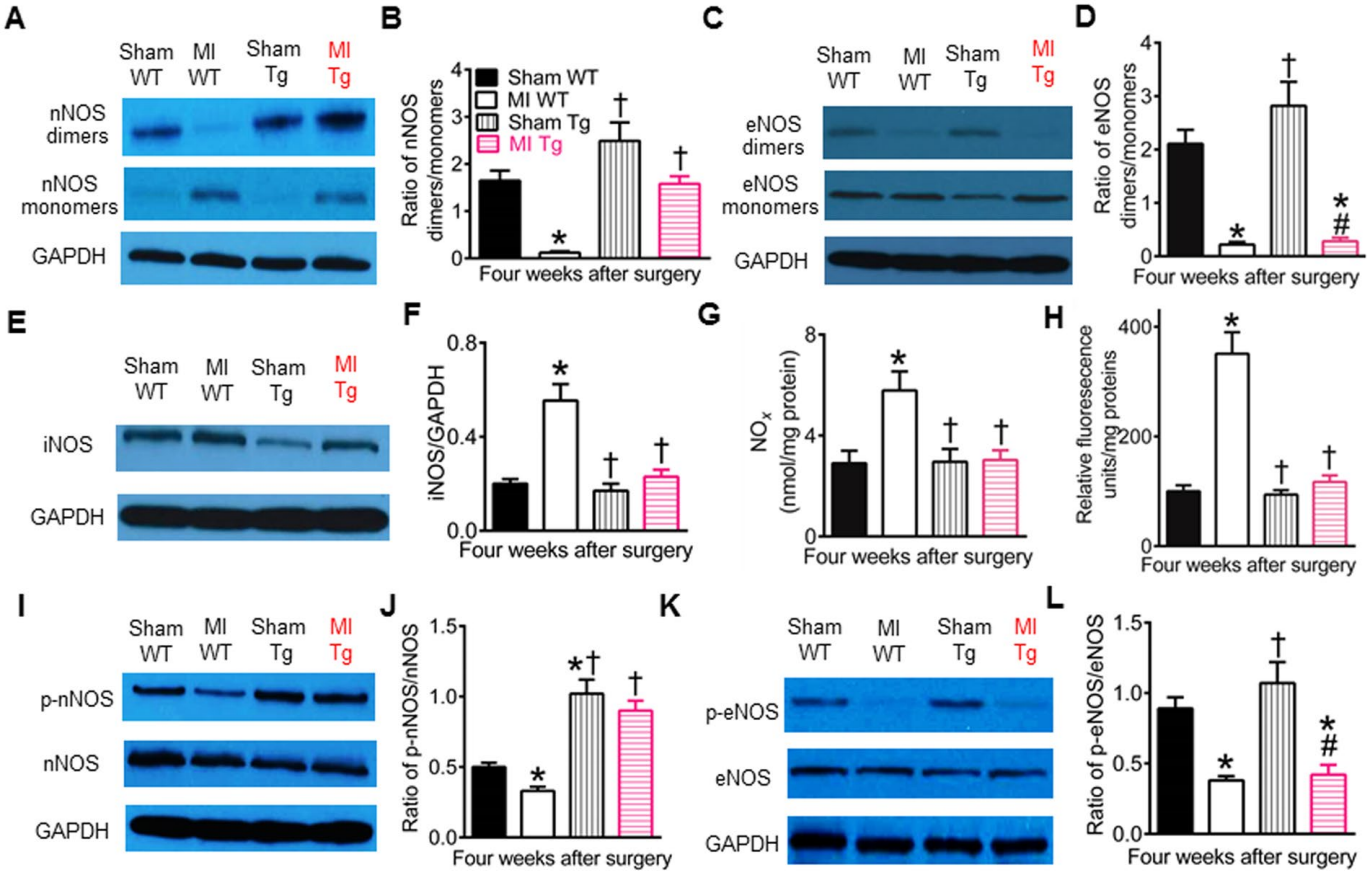
GTP cyclohydrolase 1 (GCH1) overexpression differentially regulates nitric oxide synthase (NOS) isoforms in post-infarction remodeled myocardium. (**A**) Representative Western blot bands showing the expression of dimeric and monomeric neuronal NOS (nNOS) and glyceraldehyde 3-phosphate dehydrogenase (GAPDH) as loading control in wild-type (WT) and transgenic (Tg) mice 4 weeks after myocardial infarction (MI) or sham surgery (sham); (**B**) The ratio of nNOS dimers/monomers; (**C**) Representative Western blot bands of dimeric and monomeric endothelial NOS (eNOS) and GAPDH; (**D**) The ratio of eNOS dimers/monomers; (**E**) Western blot bands showing the expression of inducible NOS (iNOS) monomers and GAPDH; (**F**) iNOS monomers normalized to GAPDH; (**G**) NO_*x*_ levels; (**H**) superoxide levels; (**I**) representative Western blot bands showing the expresiion of phosphorylated nNOS (p-nNOS), nNOS, and GAPDH; (**J**) The ratio of p-nNOS/total nNOS; (**K**) Western blot bands of phosphorylated eNOS (p-eNOS), eNOS, and GAPDH; (**L**) the ratio of phosphorylated eNOS (p-eNOS)/total eNOS. *P < 0.05 versus sham WT groups; ^†^P < 0.05 versus MI WT groups; ^#^P < 0.05 versus sham Tg groups (n = 5 mice/group).

NOS dysregulation is associated with the pathogenesis of cardiac remodeling and dysfunction^47, 48^. NOS functions only in dimeric state^49^. Thus, we measured the dimers and monomers of three isoforms of NOS: nNOS, eNOS, and iNOS, in WT and Tg mouse hearts^16^. In sham WT mice, the ratios of nNOS dimers/monomers and eNOS dimers/monomers were 1.6 ± 0.2 and 2.1 ± 0.3 (n = 5 mice/group), respectively (Fig. 9A–D). Both nNOS dimers and eNOS dimers were decreased in MI WT groups, whereas both nNOS monomers and eNOS monomers were increased in MI WT groups 4 weeks after MI compared sham WT groups. The ratios of nNOS dimers/monomers and eNOS dimers/monomers were significantly lower in MI WT than sham WT groups (P < 0.05, n = 5 mice/group) (Fig. 9B and D). Interestingly, GCH1 overexpression increased dimeric nNOS in not only sham Tg but also MI Tg myocardium (P < 0.05 versus MI WT groups, n = 5 mice/group). There were no significant differences in the ratio of nNOS dimers/monomers among sham WT, sham Tg and MI Tg groups (P > 0.05). In contrast, the ratio of eNOS dimers/monomers was not significantly changed in MI Tg groups compared with MI WT groups (P > 0.05). The expression of iNOS dimers was not detected in mouse hearts. The expression of iNOS monomers was significantly greater in WT MI than sham WT groups (Fig. 9E and F). There were no significant differences in the ratio of iNOS monomers/GAPDH between sham Tg and sham WT groups. GCH1 overexpression significantly decreased the expression of iNOS monomers 4 weeks after MI. Dimeric NOS produces NO, whereas monomeric NOS generates superoxide^49^. Compared with sham WT groups, NO and superoxide levels were significantly elevated in MI WT groups but not in sham Tg and MI Tg groups (Fig. 9G and H). These results suggest that cardiac NOS is dysregulated in MI WT mice, and GCH1 overexpression elevates nNOS dimerization after MI.

Phosphorylation of NOS modulates the production of NO and superoxide from the enzyme^50, 51^. We determined the expression of phosphorylated NOS in post-infarction remodeled myocardium in WT and Tg mice. The ratios of phosphorylated nNOS (p-nNOS)/total nNOS and phosphorylated eNOS (p-eNOS)/total eNOS were significantly decreased from 0.71 ± 0.06 in sham WT group to 0.42 ± 0.04 in MI WT group and 0.86 ± 0.08 in sham WT group to 0.32 ± 0.05 in MI WT group, respectively (P < 0.05, n = 5 mice/group) (Fig. 9I, J, K and L). Compared with sham WT groups, the ratio of p-nNOS/nNOS was elevated, and the ratio of p-eNOS/eNOS was not changed in sham Tg groups. There were no significant differences between MI Tg and sham WT groups in the ratio of p-nNOS/nNOS (P > 0.05). However, the ratio of p-eNOS/eNOS was lower in MI Tg than sham WT groups (P < 0.05). Phosphorylated iNOS was not detected in myocardium of sham WT mice. These results suggest that GCH1 overexpression elevates p-nNOS expression in post-infarction remodeled myocardium.

### Survival and parametric data

Of the 106 WT and 77 Tg mice that received sham surgery, 2 Tg and 1 WT mouse died in surgery. Of the 120 WT and 96 Tg mice that underwent MI surgery, 12 WT (10.0%) and 10 Tg (10.4%) mice did not recover from their anesthesia after surgery (Fig. S3). In the WT group, 13 additional animals died within 1 week of MI because of LV rupture or acute heart failure, as judged by postmortem findings (large infarct, cardiac dilation, pleural effusion, and severe lung congestion). Similar to the WT group, 9 mice in the Tg group died within 1 week of MI. Survival after MI did not differ between Tg and WT mice (P > 0.05 between MI Tg and MI WT groups, n = 95–104 mice/group) (Fig. S3).

## Discussion

The present study identified cardiomyocyte GCH1 as a new therapeutic target for cardiac remodeling and dysfunction after MI. We demonstrated that cardiac GCH1 was degraded in post-infarction remodeled WT hearts, concomitant with increases in the thickness of interventricular septum, LV internal diameters, infarct size, interstitial fibrosis, superoxide, free Ca^2+^ in cardiomyocytes, and p-p38 MAPK; and decreases in LV anterior wall thickness, fractional shortening, + dP/dt, BH_4_, nNOS dimerization and phosphorylation, SR Ca^2+^ release, and SR Ca^2+^ handling proteins (Fig. S4). Intriguingly, each of these parameters was markedly attenuated in the Tg mice with cardiomyocyte-specific overexpression of human GCH1 gene. These results suggest that cardiomyocyte-specific overexpression of GCH1 protects SR Ca^2+^ handling through p38 MAPK-SR Ca^2+^ handling proteins and BH_4_-nNOS-SR Ca^2+^ handling proteins pathways and attenuates cardiac remodeling and dysfunction after MI.

GCH1 is constitutively expressed in cardiomyocytes, endothelial cells, and vascular smooth muscle cells^14–16, 22, 23, 52^. It catalyzes the rearrangement of GTP to 7,8-dihydroneopterin triphosphate, which is subsequently converted to BH_4_ through the sequential action of 6-pyruvoyl tetrahydrobiopterin synthase and sepiapterin reductase^12^. In the *de novo* synthesis of BH_4_, GCH1 is the rate-limiting enzyme, making it the major determinant of intracellular BH_4_ contents in multiple cells^53^. It has been well known that BH_4_ has multiple beneficial effects on NOS, including promoting and stabilizing the formation of homodimerization of the enzymatic proteins, an active form of NOS to produce NO^53, 54^. Accumulating evidence suggests that insufficient GCH1 proteins result in hypertension, cardiovascular dysfunction, and exacerbation of myocardial ischemia/reperfusion injury^13, 14, 16, 21, 22^. Thus the activity and expression of GCH1 are critical for cardiovascular health^12, 13^. In addition, BH_4_ is a key co-factor for tryptophan hydroxylase, phenylalanine hydroxylase, and tyrosine hydroxylase^25^. In humans, GCH1 gene deficiency or mutations result in hereditary progressive dystonia, also called Segawa disease^24^. The patients with Segawa dystonia have a significantly short lifespan^25^.

We demonstrated that the thickness of LV anterior wall was significantly decreased, and the thickness of interventricular septum was increased in WT mice from 2 to 12 weeks after MI. Interestingly, cardiac GCH1 proteins were also decreased from 2 to 12 weeks after MI, whereas GCH1 mRNA levels were not significantly changed. It is known that the ubiquitin-proteasome system plays a central role in degradation of proteins^55^. Our previous work and that of other investigators indicate that the activation of the 26 S proteasome increases GCH1 degradation^16, 56, 57^. It is likely that decreased GCH1 in post-infarction remodeled myocardium results from increased degradation of GCH1 by the 26 S proteasome, rather than decreased biosynthesis of GCH1.

Our echocardiographic measurements demonstrated that WT mice developed a significant LV remodeling 4 weeks after MI, displaying a decrease in anterior wall thickness and increases in the thickness of interventricular septum and LV internal diameters. These results are consistent with a previous study in mice^58^. Cardiomyocyte-specific overexpression of GCH1 significantly increases LV anterior wall thickness and decreased the thickness of interventricular septum and internal diameters after MI. Thus, cardiac overexpression of GCH1 plays a pivotal role in preventing the development of cardiac remodeling after MI.

Myocardial fibrosis contributes to abnormal cardiac remodeling, increased ventricular stiffness, and worsening LV function^59^. In Masson’s-stained heart sections, MI WT mice displayed large myocardial infarct size and a sparred ventricular septum with interstitial fibrosis, concomitant with increases in microRNA-21 levels and p-p38 MAPK. Cardiomyocyte-specific overexpression of GCH1 decreased myocardial fibrosis and p-p38 MAPK but not microRNA-21 levels after MI. Recent studies find that microRNA-21 activates fibroblasts to produce fiber through activation of p38 MAPK^35, 60^. Our results suggest that GCH1 overexpression can reduce p-p38 MAPK after MI through a non-microRNA-21-mediated mechanism.

We showed that cardiac contractility was impaired in MI WT mice, and GCH1 overexpression improves cardiac contractility after MI. To elucidate the molecular mechanisms underlying reducing cardiac contractility in MI WT mice, we determined intracellular free Ca^2+^ in cardiomyocytes. It was elevated in MI WT mice, consistent with previous studies by Prahash and Raake, *et al*.^61, 62^. These results suggest that Ca^2+^ overload occurs in cardiomyocytes isolated from WT mice. It is clear that Ca^2+^ overload impairs excitation-contraction coupling of myocytes, thereby attenuating cardiac contractility^30^. GCH1 overexpression reduced intracellular [Ca^2+^]_i_ after MI. Improved cardiac contractility by GCH1 overexpression may be attributed to reduction of Ca^2+^ overload after MI.

Despite Ca^2+^ overload in the cytoplasm, SR Ca^2+^ release was decreased in MI WT mice. These results are consistent with the previous studies in post-infarction mouse or rat hearts^41, 63^. Interestingly, GCH1 overexpression elevated SR Ca^2+^ release after MI. SR Ca^2+^ uptake is mediated by SERCA2a, whereas RyR2 controls Ca^2+^ release from the SR^43^. Therefore, we determined the expression of cardiac RyR2 and SERCA2a. Consistent with changes in SR Ca^2+^ release, both RyR2 and SERCA2a were decreased in MI WT but not MI Tg mice. Thus, elevated SR Ca^2+^ release by GCH1 overexpression after MI may arise from increases in SERCA2a and RyR2.

The effect of BH_4_ on acute I/R injury has been studied in multiple animal models and in humans. In rats or pigs, the administration of either BH_4_ before ischemia or the combination of BH_4_ and L-arginine prior to post-ischemic reperfusion significantly reduced myocardial infarct size^64, 65^. In addition to the heart, the administration of either BH_4_ or sepiapterin, the precursor of BH_4_, prior to ischemia protects the liver, kidneys, and skeletal muscle against I/R injury^66–70^. In humans, the co-administration of BH_4_ and L-arginine attenuates endothelial dysfunction induced by forearm ischemia and reperfusion in patients with coronary artery disease and type 2 diabetes mellitus^71^. Collectively, BH_4_ plays a pivotal role in protection of tissues from acute I/R injury.

In the current study, GCH1 overexpression elevated cardiac BH_4_ concentrations in both normal and post-infarction myocardium. These results are not surprising given that GCH1 is the first and rate-limiting enzyme in the *de novo* synthesis of BH_4_^12^. A growing body of research indicates that BH_4_ is a potential therapeutic target for cardiovascular disease, such as hypertension, myocardial ischemia/reperfusion injury, pressure overload-induced cardiac hypertrophy, and diabetic vascular dysfunction^13, 38, 46, 72, 73^. Despite controversy, BH_4_ is generally reported to increase eNOS coupling and phosphorylation, elevate NO production, increase tissue cGMP levels, suppress oxidative/nitrosative stress, improve intracellular Ca^2+^ homeostasis, elevates SR Ca^2+^ cycling, and inhibit inflammation^13, 38, 46, 74, 75^. It is possible that above BH_4_–mediated signaling pathways are involved in the favorable effects of GCH1 overexpression on post-infarction remodeled myocardium.

We demonstrated that cardiac remodeling after MI resulted in increases in nNOS, eNOS, and iNOS monomers. These results were consistent with the previous studies in post-infarction rat hearts^76–78^. It is noteworthy that NOS produces NO only as a homodimer, whereas NOS is not active as a monomer^79^. Thus, we determined the expression of nNOS and eNOS dimers in mouse myocardium after MI. The ratios of nNOS dimers/monomers and eNOS dimers/monomers were significantly reduced in WT mice after MI. GCH1 overexpression prevented decreases in the ratio of nNOS dimers/monomers but not eNOS dimers/monomers and increased iNOS monomers in the heart after MI. The differential regulatory effects of GCH1 overexpression on nNOS, eNOS, and iNOS in post-infarction myocardium may be related to the fact that most of nNOS and iNOS exist in cardiac myocytes, and eNOS is mainly expressed in vascular endothelial cells^28, 74, 77^.

One limitation of this study is lack of pharmacological approaches to testing the potential of GCH1 preservation to improve cardiac remodeling and dysfunction after MI. The current study demonstrated that transgenic overexpression of human GCH1 in cardiomyocytes prevented the development of LV remodeling after MI. In this genetically engineered mouse, cardiac BH_4_ levels were significantly elevated by about 25 folds in sham-operated mice. Whether pharmacological supplementation of BH_4_ can also elevate cardiac BH_4_ to similar levels in intact animals remains elusive. In addition, transgenic overexpression of GCH1 resulted in an increase in the expression of cardiac RyRs which are responsible for SR Ca^2+^ release to trigger myocyte contraction. Whether pharmacological supplementation of BH_4_ can produce such beneficial effect is unknown. Nonetheless, our current study suggests that a combination of BH_4_ agents with increased expression of SR Ca^2+^ handling proteins has a beneficial effect on cardiac remodeling after MI. It is clear that 26 S proteasome is responsible for degradation of GCH1 proteins^56, 57^. 26 S proteasome can selectively recognize specific proteins to degradation through its 19 S regulatory particles^80^. This property of the 26 S proteasome suggests that there is a possibility to develop 26 S proteasome inhibitors with specificity towards targeting GCH1^81^. Such a 26 S proteasome inhibitor may be useful in the clinical treatment of post-infarction cardiac remodeling and dysfunction.

In summary, our results demonstrate the importance of cardiac GCH1 in reducing cardiac remodeling and dysfunction and interstitial fibrosis after MI in the mouse (Fig. S4). Cardiomyocyte-specific overexpression of GCH1 elevates BH_4_ levels and nNOS dimerization and preserves intracellular Ca^2+^ handling, thereby diminishing cardiac remodeling and dysfunction after MI (Fig. S4). The present study suggests new strategies for preventing cardiac remodeling and dysfunction in patients after MI.

## Methods

### Animals

The Tg mice with cardiomyocyte-specific overexpression of human GCH1 gene on a C57BL/6 background were developed under the control of the α-myosin heavy chain promoter, as described previously^14^. The Tg mice were identified by the presence of human GCH1 gene using polymerase chain reaction (PCR) on tail-derived genomic DNA^14^. C57BL/6 WT littermates were used as controls for the Tg mice. The animal care and all experimental procedures were performed in accordance with the NIH *Guide for the Care and Use of Laboratory Animals* (Institute for Laboratory Animal Research, National Academy of Sciences, USA, 8^th^ edition, 2011), and experimental protocols were approved by the IACUC at the Medical College of Wisconsin (Milwaukee, WI, USA).

### Induction of MI

Male WT and Tg mice at the ages of 8–10 weeks were anesthetized by intraperitoneal injection of 80 mg/kg sodium pentobarbital. A left thoracotomy was performed between the 4^th^ and 5^th^ ribs^33^. The left anterior coronary artery was permanently ligated with an 8–0 silk suture near its origin between the pulmonary outflow tract and the edge of the left atrium, as described^38^.

### Transthoracic echocardiography

Animals were sedated by the inhalation of oxygen with 1.5% isoflurane. Echocardiography was performed with a VisualSonics Vevo 770 High-resolution Imaging System (Toronto, Canada) equipped with a 30 MHz transducer (Scanhead RMV 707), as described previously^37, 82^. M-mode images were recorded from the short axis 2-chamber view at the papillary muscle level.

### PCR analysis of GCH1 mRNA

The LV was homogenized at 4 °C for PCR analysis of GCH1 mRNA, as described^16^.

### Histopathological examination of mouse hearts

The hearts were sliced transversely from the apex to the basal part of the LV at 6 μm-thickness for measurements of LV morphology and interstitial fibrosis or 4 μm-thickness for measurements of myocyte cross-sectional area with the interval of 300 μm between each section^16^. All sections were stained with Masson’s trichrome. Infarct size was expressed as total infarct circumference divided by total LV circumference.

### Real-time reverse transcriptional-polymerase chain reaction analysis of microRNA-21

Heart tissues were collected from both the septum and the LV free wall in sham WT or Tg mice and from non-infarct myocardium in MI mice 4 weeks after surgery. Homogenized tissues were used in real-time quantitative reverse transcriptional-polymerase chain reaction (qRT-PCR) analysis of microRNA-21^82^.

### Isolated Langendorff-perfused hearts

Mouse hearts were quickly mounted on an isolated Langendorff apparatus and perfused retrogradely through the aorta at a constant pressure of 80 mmHg with Krebs-Henseleit buffer, as described^37, 83^. LV end-diastolic pressure was set to 5 mmHg by adjusting the volume of the intracardiac balloon. After 30 min of stabilization, LV systolic pressure and + dP/dt were determined^37^. The intracardiac balloon volume was set at zero volume, inflated to 20 µl, and subsequently increased in 5-µl intervals using an air-tight Hamilton syringe until 70 µl to obtain LV end-diastolic pressure-volume relationship^84^.

### Measurements of cardiomyocyte [Ca^2+^]_i_

Cardiomyocytes isolated from mice 4 weeks after MI or sham surgery were loaded with Fura-2 AM (Fura-2 acetoxymethyl ester) at a concentration of 5 µM, as described^16^. The cells were stimulated in an electric field at 0.5 Hz for 30 s followed by a period of 20 s without stimulation and continuously perfused with a solution containing 1.0 mM CaCl_2_ without and with 20 nM isoproterenol^85^. The ratio of the emitted fluorescence at the two excitation wavelengths (340/380 nM ratio) was calculated to provide an index of intracellular [Ca^2+^]_i_.

### Measurements of SR Ca^2+^ release

SR Ca^2+^ release was induced by rapid application of 10 mM caffeine to the cells in the presence of 0 Na^+^ and 0 Ca^2+^ Tyrode buffer to inhibit Na^+^-Ca^2+^ exchange, as described^16^. The protocol was repeated after 100 s exposure to 20 nM isoproterenol added to Tyrode buffer.

### Western blot analysis

The LV homogenates that contained 50 µg of protein were applied to 7.5% sodium dodecyl sulfate (SDS)-polyacrylamide gel and subjected to immunoblot analysis by incubation with primary antibodies against human GCH1, mouse GCH1, p38 MAPK, p-p38 MAPK (tryptophan180/tyrosine 182), nNOS, eNOS, iNOS, RyR2, SERCA2a, T-PLB, p-PLB at serine 16, and GAPDH at 4 °C, as described^82, 86^. The membrane was then incubated with the appropriate anti-mouse or anti-rabbit secondary antibody. Immunoreactive bands were visualized by enhanced chemiluminescence followed by densitometric analysis using image acquisition and analysis software (Image J, National Institutes of Health, Baltimore, MD, USA).

### Assay of BH_4_

BH_4_ was quantified in LV tissue homogenates by HPLC with electrochemical detection (ESA Biosciences CoulArray® system Model 542, Chelmsford, MA, USA)^75, 83^. Authentic BH_4_ solutions (10–100 nM) were used as standards, and sample concentrations were normalized to protein content measured by the bicinchoninic acid protein assay.

### Measurements of NO and O_2_^•−^

Tissue NO and its metabolite products (nitrate and nitrite) in the supernatant, collectively known as NO_*x*_, were assayed using a NO chemiluminescence analyzer (Siever 280i NO Analyzer)^14^. Lucigenin, a compound that emits light upon interaction with O_2_^•−^, was used to quantify the O_2_^•−^ production from myocardium^16^. The data were presented in relative light units (RLUs) per mg protein.

### Statistics

All data are expressed as mean ± S.E.M. Statistical analysis was performed with one-way ANOVA followed by Bonferroni *post-hoc* test for multiple comparisons of multiple group means or with Student’s *t*-test for comparisons between two group means. Repeated-measures ANOVA was used to compare the differences in heart surface area, echocardiographic parameters, GCH1 mRNA, GCH1 proteins, and LV end-diastolic pressure-volume relationships at different time points. A value of P < 0.05 was considered statistically different.

### Data availability

The data that support the findings in this study are available from the corresponding author on request.

## Electronic supplementary material

Supplement
