# Supplementary material for: Transgenic overexpression of GTP cyclohydrolase 1 in cardiomyocytes ameliorates post-infarction cardiac remodeling
Source: Sci Rep. 2017 Jun 8;7:3093. doi: 10.1038/s41598-017-03234-6 (PMC5465102; doi:10.1038/s41598-017-03234-6)
Supplement: Supplementary file 1 — Supplement [file 41598_2017_3234_MOESM1_ESM.pdf]

Supplementary Materials for

Transgenic overexpression of GTP cyclohydrolase 1 in cardiomyocytes ameliorates post-infarction cardiac remodeling

Yanan Liu, Shelley L. Baumgardt, Juan Fang, Yang Shi, Shigang Qiao, Zeljko J. Bosnjak, Jeannette Vásquez-Vivar, Zhengyuan Xia, David C. Warltier, Judy R. Kersten, Zhi-Dong Ge*

*Corresponding author. E–mail: Wilson.ge99@gmail.com (Z.-D.G.)

The Supplementary Materials includes:

Supplementary materials and methods

Table S1. Time-dependent changes in body weight and heart rate in C57BL/6 mice after surgery.

Table S2. Physiological and left ventricular hemodynamic parameters in C57BL/6 and Tg mice 4 weeks after myocardial infarction.

Figure S1. Myocardial responsiveness to β-adrenergic stimulation *in vivo* in mice 4 weeks after myocardial infarction or sham surgery

Figure S2. 7-nitroindazole blocked the effects of GCH1 overexpression on post-infarction remodeled hearts.

Figure S3. Survival curves.

Figure S4. Potential mechanisms.

Material and Methods

Animals

The Tg mice with cardiomyocyte-specific overexpression of human GCH1 gene on a C57BL/6 background were developed under the control of the α-myosin heavy chain promoter, as described previously[1]. The Tg mice were identified by the presence of human GCH1 gene using polymerase chain reaction (PCR) on tail-derived genomic DNA[1]. C57BL/6 WT littermates were used as controls for the Tg mice. The animals were kept on a 12-h light-dark cycle in a temperature-controlled room and received standard rodent maintenance diet and water ad libitum.

Study approval

The animal care and all experimental procedures were performed in accordance with the NIH *Guide for the Care and Use of Laboratory Animals* (Institute for Laboratory Animal Research, National Academy of Sciences, USA, 8th edition, 2011), and experimental protocols were approved by the IACUC at the Medical College of Wisconsin (Milwaukee, WI, USA) (animal use approval numbers 689, 2484, and 4555).

Induction of myocardial infarction (MI)

Male WT and Tg mice at the ages of 8-10 weeks were anesthetized by intraperitoneal injection of 80 mg/kg sodium pentobarbital. The trachea was cannulated with a polyethylene 60 tube connected to a positive-pressure mouse respirator (MiniVent type 845; Hugo Sachs ElectroniK-Harvard Apparatus, Hugstetten, Germany) with a tidal volume of 250 μl[2]. The mice were ventilated with room air supplemented with 100% oxygen at approximately 102 breaths per minute. A left thoracotomy was performed between the 4th and 5th ribs, and the lungs retracted to expose the heart[3]. After the pericardial tissue was removed, the left anterior coronary artery was visualized under a microscope and permanently ligated with an 8-0 silk suture near its origin between the pulmonary outflow tract and the edge of the left atrium, as described[4]. The

lungs were inflated by increasing positive end-respiratory pressure. The chest wall was closed in 2 layers: rib-muscle and skin. Animals were kept in a warm chamber heated by both a heating pad below the chamber and a heating lamp over the chamber until recovery. Sham-operated animals underwent the same procedure except the coronary artery ligation. Rectal temperature was monitored and maintained between 36.8 and 37.3 ^{o}C by a TC-1000 Temperature controller (CWE Inc., Ardmore, PA, USA) throughout surgical procedure. For analgesia, buprenorphine (0.05 mg/kg) was administered subcutaneously immediately after the chest closure and every 8 h for the next 48 h.

Transthoracic echocardiography

Non-invasive transthoracic echocardiography was used to evaluate left ventricular geometry and function in WT and Tg mice at 0 (baseline), 1, 2, 4, 8, and 12 weeks after MI or sham surgery. Animals were sedated by the inhalation of oxygen with 1.5 % isoflurane. Echocardiography was performed with a VisualSonics Vevo 770 High-resolution Imaging System (Toronto, Canada) equipped with a 30 MHz transducer (Scanhead RMV 707), as described previously[5,6]. M-mode images were recorded from the short axis 2-chamber view at the papillary muscle level. Heart rate, the thickness of anterior wall and interventricular septum of the left ventricle (LV), and LV end-diastolic and systolic diameters were measured. Fractional shortening was calculated using the following equation: % factional shortening = (LV end-diastolic diameter–LV end-systolic diameter)/LV end-diastolic diameter×100.

Polymerase chain reaction (PCR) analysis of GCH1 mRNA

To examine time-dependent changes in GCH1 mRNA levels, C57BL/6 mouse hearts were excised at 0 (baseline), 1, 2, 4, 8, and 12 weeks after surgery and snap frozen in liquid nitrogen immediately after excision. The LV was homogenized at 4 ^{o}C for PCR analysis of GCH1 mRNA, as described.[7] Briefly, total RNA was extracted from snap frozen tissue in 1 ml of

TRIzol reagent (Invitrogen, Carlsbad, CA, USA) per 100 mg of heart tissue according to the protocol of the manufacturer. Genomic DNA was digested by treatment with RNase-free DNase (Ambion, Austin, TX, USA), and RNA concentrations were determined spectrophotometrically. Complementary DNA was synthesized from 1 μg of total RNA and random hexamer primers by using SuperScript III first-strand synthesis system for PCR (Invitrogen) according to the manufacturer's directions, as described[1]. Complementary DNA was used in PCR with the following primers for various *GCH1* mRNA transcripts: human (transgenic): forward 5'-CGCCTACTCGTCCATCCTGA-3', reverse 5'- CCTTCACAATCACCATCTCA-3' (product size 181 bp); mouse (endogenous): forward 5'- TGCTTACTCGTCCATTCTGC-3', reverse 5'-CCTTCACAATCACCATCTCG-3' (product size 181 bp). GAPDH was used as an internal control for all experiments. PCR reactions were performed in a 25-μl volume containing 1 μl of complementary DNA, 25 pmol of sequence-specific primers, and 22.5 μl of Platinum PCR SuperMix High Fidelity (Invitrogen). A 10-μl aliquot of the PCR product was resolved by 1% Tris acetate-EDTA-agarose gel electrophoresis, and densitometric analysis of specific bands was performed using Alpha Imager (Alpha Innotech, San Leandro, CA, USA). Relative mRNA level was determined as 2[(Ct/GAPDH−Ct/gene of interest)]. The results are presented as -fold expression normalized to GAPDH.

Measurements of blood pressure and intracardiac hemodynamics

Pentobarbital-anesthetized mice were connected to a mouse ventilator after endotracheal intubation, as described in the section of "Induction of MI". After bilateral vagotomy, the right carotid artery was cannulated with a micromanometer-tipped mouse pressure catheter (SPR-671, Millar Instruments, Houston, TX, USA)[7]. The catheter was connected to an ADInstrument pressure transducer (MLT0380/D, ADInstruments, Colorado Springs, CO, USA) and a Powerlab data acquisition system (ADInstruments). After a 30 min of stabilization, blood pressure was continuously recorded for 20 min. The catheter was then advanced retrograde into the left

ventricle (LV) for recording of LV pressure[8]. Steady-state LV pressure was used to analyze off-line for the derivation of traditional parameters of LV systolic (peak LV pressure and +dP/dt) and diastolic (end-diastolic pressure, -dP/dt, and Tau (τ), as described.[9] In the sunsets of animals from each group, intravenous isoproterenol (a β_1- and β_2-adrenergic agonist) at the concentrations of 0.1, 1.0, 10.0, and 100 pg/g body weight was administered to assess systolic and diastolic function of the LV under β-adrenergic stimulation. At the end of the experiment, animals were euthanized, and heart, LV, and lung were weighed. Heart weight, left ventricular weight, and lung weight were normalized to body weight.

Histopathological examination of mouse hearts

Pentobarbital-anesthetized mice received intracardiac saturated KCl (0.1 ml) to arrest the heart in diastole. The hearts were washed with cold phosphate-buffered saline, fixed with 4% paraformaldehyde, dehydrated, and embedded in paraffin. The hearts were sliced transversely from the apex to the basal part of the LV at 6 μm-thickness for measurements of LV morphology and interstitial fibrosis or 4 μm-thickness for measurements of myocyte cross-sectional area with the interval of 300 μm between each section[7]. All sections were mounted on glass slides and stained with Masson's trichrome. Images were captured with a SPOT Insight™ camera with the use of SPOT imaging software (Diagnostic Instruments, Inc., Sterling, MI, USA)[1,5]. SPOT software was used to measure the circumference of the infarcted region and the LV. Total LV circumference was calculated as the sum of endocardial and pericardial segment lengths from all sections. Infarct size was expressed as total infarct circumference divided by total LV circumference. The LV internal diameters were measured with SPOT software at mid-ventricular level. Fibrosis areas within Masson's trichrome-stained sections were measured by visualizing blue-stained areas, exclusive of staining that colocalized with perivascular or intramural vascular structures, the endocardium, or LV trabeculae using SPOT software. The

percentage of total fibrosis area was calculated as the summed blue-stained areas divided by total ventricular area. To determine myocyte cross-sectional area, the remote zone and border zone of Masson's trichrome-stained sections were imaged with a Nikon microscope using a 20× objective. A minimum of 100 myocytes from five different animals was quantified for each experimental group.

Real-time reverse transcriptional-polymerase chain reaction analysis of microRNA-21

Heart tissues were collected from both the septum and the LV free wall in sham WT or Tg mice and from non-infarct myocardium in MI mice 4 weeks after surgery. Homogenized tissues were used in real-time quantitative reverse transcriptional-polymerase chain reaction (qRT-PCR) analysis of microRNA-21[6].

Total RNA from heart tissues was extracted using Qiazol reagent according to the protocol of the manufacturer (Qiagen, Valencia, CA, USA). Chloroform was added and samples were centrifuged to facilitate phase separation. The aqueous phase was extracted and combined with ethanol in miRNeasy Mini spin columns (Qiagen). Total RNA was eluted in RNase-free water. The concentration of extracted total RNA was quantified by the Epoch spectrophotometer (Biotek, Winooski, VT, USA). Samples were considered pure if the A260/280 ratio was between 1.9 and 2.0. One µg of total RNA from each sample was used to generate cDNA using miScript Reverse transcriptase mix, nucleic mix, and HiFlex Buffer (Qiagen). The complementary DNA product was measured in triplicate using miScript Primer Assays for miR-21 (Qiagen). qRT-PCR was conducted using the BioRad iCycler Real-Time PCR Detection System. Expression of miR-21 was normalized by expression of the housekeeping gene Rnu-6 (Qiagen). The relative gene expressions were calculated in accordance with the ΔΔCt method. Relative miRNA levels were expressed as percentages of sham WT groups.

Isolated Langendorff-perfused hearts

Under anesthesia of sodium pentobarbital, mouse hearts were quickly excised at body temperature, placed immediately to ice cold Krebs-Henseleit buffer, mounted on an isolated Langendorff apparatus, and perfused retrogradely through the aorta at a constant pressure of 80 mmHg with Krebs-Henseleit buffer containing (in mM) NaCl 118, $NaHCO_3$ 25, KCl 4.7, $MgCl_2$ 1.2, $CaCl_2$ 2.5, KH_2PO_4 1.2, EDTA 0.5, and glucose 11, as described[5,10]. The buffer was continuously bubbled with a mixture of 95% oxygen/5% carbon dioxide via an in-line filter (5 µm pore size). A fluid-filled plastic balloon was inserted into the chamber of the LV via the mitral valve and connected to a pressure transducer for continuous measurement of LV pressure. The hearts were immersed in perfusate maintained at 37.2 ± 0.3 ^{o}C. Coronary flow was monitored by an in-line flow probe connected to a flow meter (Transonics Systems Inc., Ithaca, NY, USA). The LV pressure signal was monitored to obtain LV end-systolic and end-diastolic pressure.

LV contractile performance

LV end-diastolic pressure was set to 5 mmHg by adjusting the volume of the intracardiac balloon. After 30 min of stabilization, LV systolic pressure and +dP/dt were determined[5].

LV end-diastolic pressure-volume relationship

The intracardiac balloon volume was set at zero volume, and the heart was stabilized for 30 min. The balloon volume was inflated to 20 µl and subsequently increased in 5-µl intervals using an air-tight Hamilton syringe until 70 µl[11]. LV functional measurements were obtained 2 min after each increment in volume when a new steady state was reached.

Isolation of cardiomyocytes

Cardiomyocytes were isolated from adult C57BL/6 or Tg mice 4 weeks after MI or sham surgery, as described[7]. Briefly, mouse hearts were cannulated via the aorta onto a blunted 20

gauge needle and retrogradely perfused for 10 min at 37 ^{o}C with oxygenated solution A: 130 mM NaCl, 25 mM N-2-hydroxyethylpiperazine-N$^{/}$-2-ethanesulfonic acid (HEPES), 22 mM glucose monohydrate, 5.4 mM KCl, 0.5 mM $MgCl_2$, 0.4 mM NaH_2PO_4 (pH 7.4). The heart was then then perfused with 37°C oxygenated solution A supplemented with ~200 U/ml collagenase Type–II (Worthington, NJ, USA) and 0.08 mM Ca^{2+} for 20 min. An incision was made in viable myocardium to assure that the BZ proximal to the infarction was excluded. In sham hearts, cardiomyocytes were harvested from both the septum and the LV free wall. The viable LV free wall and septum were chopped into small pieces and gently stirred in solution A supplemented with 15 µM bovine serum albumin (Sigma Aldrich, St. Louis, MO, USA) and ~100 U/ml deoxyribonuclease I (Worthington)[12]. In MI hearts, the infarcted area and a 2-mm rim of peri-infarct tissue were carefully removed and discarded. The remaining non-infarct myocardium was dissected free from the atria and repeatedly passed through a plastic transfer pipette to disaggregate the cells into a single-cell suspension. Subsequently, myocytes were enriched by sedimentation in perfusion buffer containing 5% bovine calf serum while slowly exposing the cells to increasing concentrations of $CaCl_2$ to achieve a final concentration of 1.2 mM. The final cell pellet containing calcium-tolerant myocytes was resuspended in the culture media containing Hanks' salts, 2 mM L-glutamine, 5% bovine calf serum, 10 mM 2,3-butanedione monoxime, and 100 U/ml penicillin. After isolation, the myocytes were stored in Tyrode solution (in mM: 132 NaCl, 10 HEPES, 5 glucose, 5 KCl, 1.0 $CaCl_2$, 1.2 $MgCl_2$; adjusted to pH 7.4). Experiments were conducted at room temperature within 6 h after isolation using Tyrode solution.

Measurements of cardiomyocyte $[Ca^{2+}]_i$

Cardiomyocytes were loaded with a fluorescent dye Fura-2 AM (Fura-2 acetoxymethyl ester) (Molecular Probes) at a concentration of 5 µM and plated on laminin-coated coverslip mounted

in a perfusion chamber for 5 min for the de-esterification of the fluorescent probes[7]. After loading, myocytes were centrifuged, washed with solution B contained (in mM) 140 NaCl, 5.4 KCl, 0.5 $MgCl_2$, 1.0 $CaCl_2$, 5.5 glucose, 0.4 NaH_2PO_4, 5.0 HEPES (pH 7.4) to remove extracellular Fura-2 and left for 30 min to ensure complete hydrolysis of the intracellular ester. To measure intracellular $[Ca^{2+}]_i$, the cells were stimulated in an electric field at 0.5 Hz for 30 s followed by a period of 20 s without stimulation and continuously perfused with solution B containing 1.0 mM $CaCl_2$ without (baseline) and with 20 nM isoproterenol (Sigma-Aldrich)[13]. The resulting fluorescence emitted at 510 nm was recorded by a dual-excitation fluorescence photomultiplier (IonOptix LLC, Westwood, MA, USA), and the ratio of the emitted fluorescence at the two excitation wavelengths (340/380 nM ratio) was calculated to provide an index of intracellular $[Ca^{2+}]_i$. Basal $[Ca^{2+}]_I$ and the height and T50 decay of the Ca^{2+} transient were measured in electrically stimulated (0.5 Hz) myocytes.

Measurements of SR Ca^{2+} release

Cardiomyocytes were loaded with Fura-2 AM with Pluronic F-127 (0.04%) to aid dispersion followed by 30 min of de-esterization at 22 ^oC before recordings. Resting Ca^{2+} measurements were taken from the quiescent cells for 3-4 min prior to the application of any agonists. SR Ca^{2+} release was assessed by rapid application of 10 mM caffeine to the cells to induce SR Ca^{2+} release in the presence of 0 Na^+ and 0 Ca^{2+} Tyrode buffer to inhibit Na^+-Ca^{2+} exchange, as described[7]. The protocol was repeated after 100 s exposure to 20 nM isoproterenol added to Tyrode buffer. Caffeine-induced Ca^{2+} release was recorded by a dual-excitation fluorescence photomultiplier. Basal $[Ca^{2+}]_I$ and the height and T50 decay of the Ca^{2+} transient were measured in electrically stimulated (0.5 Hz) myocytes.

Western blot analysis

The LV was harvested in WT and Tg mice 4 weeks after surgery and homogenized in a buffer containing 20.0 mM 3-(N-morpholino)propanesulfonic acid (MOPS), 2.0 mM EGTA, 5.0 mM EDTA, protease inhibitor cocktail (1:100; Calbiochem, San Diego, CA, USA), phosphatase inhibitors cocktail (1:100; Calbiochem), 0.5% detergent (Nonidet™ P-40 detergent pH 7.4, Sigma-Aldrich). Immunoblots were performed using standard techniques, as described[6,14]. Briefly, tissue homogenates that contained 50 µg of protein were applied to 7.5% sodium dodecyl sulfate (SDS)-polyacrylamide gel and subjected to immunoblot analysis by incubation with a 1:1,000 dilution of primary antibody against human GCH1 (LifeSpan BioSciences, Inc., Seattle, WA, USA), mouse GCH1 (BD Biosciences, San Jose, CA, USA), p38 mitogen-activated protein kinase (p38 MAPK, Cell Signaling Technology, Danvers, MA), phosphorylated p38 MAPK (p-p38 MAPK) (tryptophan180/tyrosine 182) (Cell Signaling Technology), neuronal nitric oxide synthase (nNOS, Invitrogen), phosphorylated nNOS (p-nNOS) (serine 1412, Affinity Bioreagents, Golden, CO), endothelial nitric oxide synthase (eNOS, Santa Cruz Biotechnologies, Santa Cruz, CA), phosphorylated eNOS (p-eNOS) (serine 1177, Calbiochem), inducible nitric oxide synthase (iNOS, Abcam, Cambridge, MA, USA), ryanodine receptors (RyR2, EMD Millipore, Billerica, MA, USA), sarcoplasmic reticulum Ca^{2+} ATPase (SERCA2a, Abcam), or phospholamban (PLB, Abcam) at 4°C. The membrane was washed and then incubated with a 1:2000 dilution of anti-mouse (ThermoFisher Scientific, Waltham, MA, USA) or anti-rabbit secondary antibody (BioRad Laboratories, Inc., Hercules, CA, USA). The normal function of NOS to generate NO requires the dimer of the enzyme proteins[15,16]. To investigate the formation of NOS dimers in the myocardium, non-boiled protein lysate was resolved by 6% SDS-PAGE at 4°C overnight[10,14]. Membranes were incubated with a 1:2000 dilution of mouse anti-nNOS or anti-eNOS monoclonal antibodies (BD Transduction Laboratories, San Jose, CA). Immunoreactive bands were visualized by enhanced chemiluminescence followed by densitometric analysis using image acquisition and analysis software (Image J, National Institutes of Health, Baltimore, MD, USA).

Assay of tetrahydrobiopterin

C57BL/6 and Tg mouse hearts were rapidly excised 4 weeks after Mi or sham surgery. Tetrahydrobiopterin (BH_4) was quantified in LV tissue homogenates by HPLC with electrochemical detection (ESA Biosciences CoulArray® system Model 542, Chelmsford, MA, USA)[10,17]. Filtrates were analyzed on a HPLC system (ESA Biosciences CoulArray® system, Model 582 and 542) using an analytical Polar-RP column eluted with argon saturated 50 mM phosphate buffer (pH 2.6). Authentic BH_4 solutions (10-100 nM) were used as standards, and sample concentrations were normalized to protein content measured by the bicinchoninic acid protein assay.

Measurements of NO and $O_2^{\bullet-}$

Tissue NO and its metabolite products (nitrate and nitrite) in the supernatant, collectively known as NO_x, were assayed using a NO chemiluminescence analyzer (Siever 280i NO Analyzer)[1]. Lucigenin, a compound that emits light upon interaction with $O_2^{\bullet-}$, was used to quantify the $O_2^{\bullet-}$ production from myocardium[7]. The data were presented in relative light units (RLUs) per mg protein. Relative $O_2^{\bullet-}$ levels were expressed as percentages compared to WT controls.

Treatment of Tg mice with 7-nitroindazole

Tg mice were subjected to permanent ligation of the left coronary artery to produce infarction, and sham-operated mice underwent the same surgical procedure except coronary artery ligation. At 3 days after surgery, Tg mice were given 10 mg/kg/day 7-nitroindazole (7-NI) (Cayman Chemicals) for 4 weeks using a plastic feeding tube (Instech Laboratories, Inc., Plymouth Meeting, PA, USA) or the vehicle, 1% Tween-80-saline solution, as control[18]. The dimensions of the LV were assessed with an echocardiography at baseline and four weeks after the treatment of the mice with 7-NI. The LV end-diastolic pressure-volume relationship was

analyzed in Langendorff-perfused mouse hearts, as described in the section of "Determination of cardiac function in isolated hearts".

Statistics

All data are expressed as mean ± S.E.M. Statistical analysis was performed with one-way ANOVA followed by Bonferroni *post-hoc* test for multiple comparisons of multiple group means or with Student's *t*-test for comparisons between two group means. Repeated-measures ANOVA was used to compare the differences in heart surface area, echocardiographic parameters, GCH1 mRNA, GCH1 proteins, and LV end-diastolic pressure-volume relationships at different time points. A value of $P<0.05$ was considered statistically different.

Results

Body weight and heart rate were not changed in WT mice after MI

Body weight and heart rate were comparable between MI WT and sham WT groups throughout the experiment ($P>0.05$, n=8-10 mice/group) (Table S1).

GCH1 overexpression improved myocardial responsiveness to β-adrenergic stimulation after MI *in vivo*

Abnormalities in myocardial β-adrenoceptor signaling are implicated in cardiac remodeling and dysfunction after MI[12,19,20]. We determined myocardial responsiveness to the β1 and β2-adrenergic agonist isoproterenol *in vivo* in Tg and WT mice 4 weeks after MI or sham surgery. Blood pressure and intracardiac hemodynamics were measured with a pressure conductance catheter. There were no significant differences in LV systolic pressure, LV diastolic pressure, the rate of LV pressure rise (+dP/dt), the rate of LV pressure decrease (-dP/dt), and time constant of left ventricular relaxation Tau (τ) values among 4 experimental groups without

intravenous administration of isoproterenol (P>0.0083, n=8-10 mice/group) (Table S2). In all mice, intravenous infusion of isoproterenol at the concentrations of 0.1, 1.0, 10.0, and 100.0 pg/g body weight resulted in gradual increases in LV developed pressure and ±dP/dt (Figure S1). No differences existed between sham Tg and sham WT groups at various concentrations of isoproterenol (P>0.05, n=6-7 mice/group). Either 10.0 or 100.0 pg/g isoproterenol-elicited increases in LV developed pressure and ±dP/dt were significantly attenuated in MI WT groups compared with sham WT groups (P<0.0083). Interestingly, GCH1 overexpression significantly elevated 10.0 and 100.0 isoproterenol-induced increases in LV developed pressure and ±dP/dt after MI (P<0.0083 between MI Tg and MI WT groups). These results indicate that myocardial responsiveness to β-adrenergic stimulation is elevated in Tg mice after MI.

Inhibition of nNOS blocked the beneficial effects of GCH1 overexpression on cardiac remodeling

To examine the role of nNOS in the effect of GCH1 overexpression on post-infarction cardiac remodeling, we used 10 mg/kg/day 7-nitroindazole (7-NI, a specific inhibitor for nNOS) to treat Tg mice for 4 weeks 3 days after MI or sham surgery. The thickness of LV anterior and posterior walls and fractional shortening were comparable among 4 groups at baseline (P>0.05, n=8-12 mice/group) (Figure S2). The treatment of sham Tg mice with 7-NI for 4 weeks (7-NI Tg group) did not significantly change LV wall thickness and fractional shortening (P>0.05). Compared with sham Tg groups, there were no significant differences in LV dimensions and fractional shortening in MI Tg group (P>0.05). Interestingly, the treatment of MI Tg mice with 7-NI (MI Tg+7-NI group) resulted in significant decreases in LV anterior wall thickness at both end diastole and end systole and fractional shortening and a significant increase in posterior wall thickness at both end diastole and end systole (P<0.0083, n=8-12 mice/group). In isolated Langendorff-perfused hearts, LV end-diastolic pressure was comparable among sham Tg, 7-NI

Tg, and MI Tg groups at 20, 25, 30 µl of LV volume (P>0.05). Compared with sham Tg, 7-NI Tg, or MI Tg groups, the LV end-diastolic pressure was significantly decreased in MI Tg+7-NI groups from 40 to 70 µl of LV volume (P<0.0083, n=8-12 mice/group). These results suggest that the inhibition of nNOS activity with 7-NI in MI Tg mice exacerbates cardiac remodeling and dysfunction after MI.

Table S1. Time-dependent changes in body weight and heart rate in C57BL/6 wild-type (WT) mice after surgery

Weeks after surgery	Body weight, g		P values	Heart rate, beats/min		P values
	Sham WT	MI WT		Sham WT	MI WT	
0	25.8±0.4	25.9±0.4	NS	457±18	475±18	NS
1	26.3±0.3	26.4±0.4	NS	452±17	468±15	NS
2	26.9±0.4	27.1±0.6	NS	447±15	485±17	NS
4	27.6±0.4	27.2±0.6	NS	465±16	477±20	NS
8	29.0±0.5	28.2±0.6	NS	458±16	485±20	NS
12	30.1±0.6	28.6±0.6	NS	469±17	496±20	NS

MI, myocardial infarction; NS, not significant.

Table S2. Physiological and left ventricular hemodynamic parameters in C57BL/6 wild-type (WT) and GCH1 transgenic (Tg) mice 4 weeks after surgery

	Sham WT	MI WT	Sham Tg	MI Tg
Body weight, g	27.8±0.5	27.0±0.4	27.6±0.5	27.9±0.6
Hear rate, beats/minute	384±17	404±19	393±15	388±15
Mean arterial blood pressure, mmHg	104±9	87±10	101±9	92±8
LV systolic pressure, mmHg	109±4	89±7	107±5	101±6
LV diastolic pressure, mmHg	5.8±0.9	8.6±1.6	5.4±0.7	7.6±1.0
+dP/dt, mmHg/s	6602±732	4381±641	6436±579	6058±663
-dP/dt, mmHg/s	5128±695	3299±332	5060±500	4589±526
τ, ms	7.2±0.5	8.8±0.6	7.4±0.5	7.8±0.6
Heart weight, mg	129±5	168±8*	120±4†	136±8
LV weight, mg	104±5	136±8*	96±5†	108±7
Lung weight, mg	157±5	232±15*	159±7†	180±8
Heart/body weight, g	4.6±0.2	6.2±0.3*	4.4±0.2†	4.9±0.3†
LV/body weight, mg/g	3.7±0.2	5.0±0.2*	3.5±0.2†	3.9±0.3†
Lung/body weight, mg/g	5.7±0.2	8.6±0.5*	5.8±0.2†	6.5±0.3†

+dP/dt, the rate of left ventricular pressure rise; -dP/dt, the rate of left ventricular pressure decrease; LV, left ventricle; MI, myocardial infarction; ms, millisecond; Tau (τ), time constant of LV relaxation. Blood pressure and left ventricular hemodynamics were measured with a Millar Mikro-tip pressure transducer catheter. One-way ANOVA followed by Bonferroni *post-hoc* test was used to evaluate the differences among groups. Six *post-hoc* tests were performed, and a value of P<0.0083 was considered statistically different. *P<0.0083 versus sham WT groups; †P<0.0083 versus MI WT groups (n=8-10 mice/group).

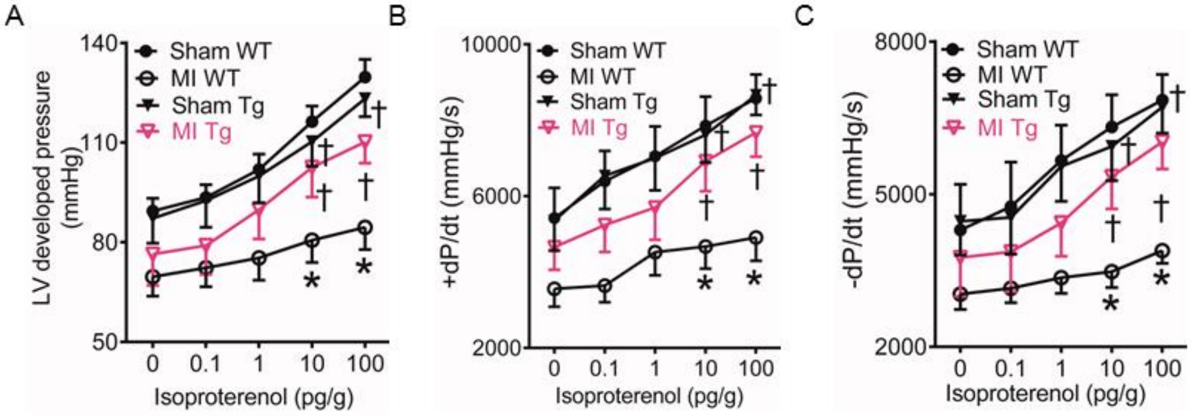

Figure S1 GTP cyclohydrolase 1 (GCH1) overexpression elevated myocardial responsiveness to β-adrenergic stimulation *in vivo* after myocardial infarction (MI). A: left ventricular (LV) developed pressure; B: the maximum rate of LV pressure rise (+dP/dt); C: the maximumrate of LV pressure decrease (-dP/dt). LV systolic and diastolic pressure was assessed with a

conduction catheter in wild-type (WT) and transgenic (Tg) GCH1 mice 4 weeks after MI or sham surgery in the presence and absence of intravenous infusion of isoproterenol. One-way ANOVA followed by 6 Bonferroni *post-hoc* tests was used to evaluate the differences among 4 groups. *P<0.0083 (an adjusted P value for statistical difference due to 6 *post-hoc* tests in 4 groups) versus sham WT groups; †P<0.0083 versus MI WT groups (n=6-7 mice/group).

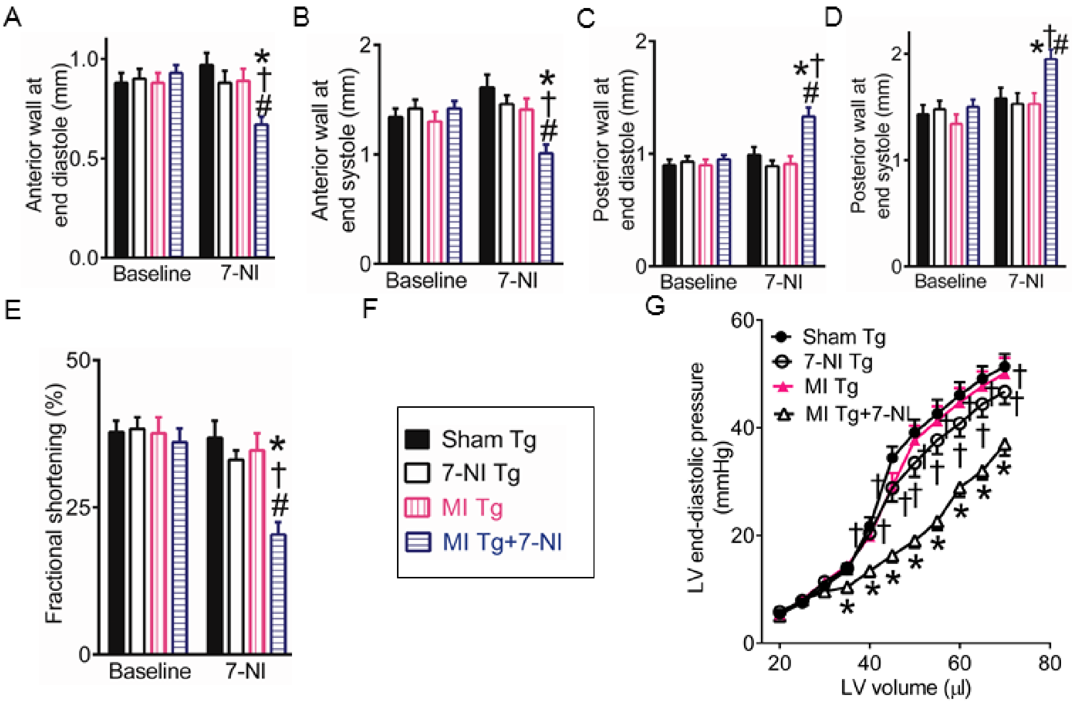

Figure S2 7-nitroindazole (7-NI) blocked the effects of GTP cyclohydrolase 1 (GCH1) overexpression on post-infarction remodeled hearts. A: anterior wall thickness at end diastole; B: anterior wall thickness at end systole; C: posterior wall thickness at end diastole; D: posterior wall thickness at end systole: E: fractional shorteining; F: figure legend for figures A, B, C, D, and E. Transgenic (Tg) GCH1 mice were orally given 7-NI for 4 weeks, starting at 3 days after myocardial infarction (MI) or sham surgery. Echocardiography was used to mesuare the thickness of left ventricular wall and fractional shortening. G: the left ventricular end-diastolic

pressure-volume relationship in Langendorff-perfused hearts. The left ventricular end-diastolic pressure-volume relationship was determined in isolated hearts. *P<0.0083 (an adjusted P value for statistical difference due to 6 *post-hoc* tests in 4 groups) versus sham Tg groups; †P<0.0083 versus 7-NI Tg groups, #P<0.0083 versus MI Tg groups (n=8-12 mice/group).

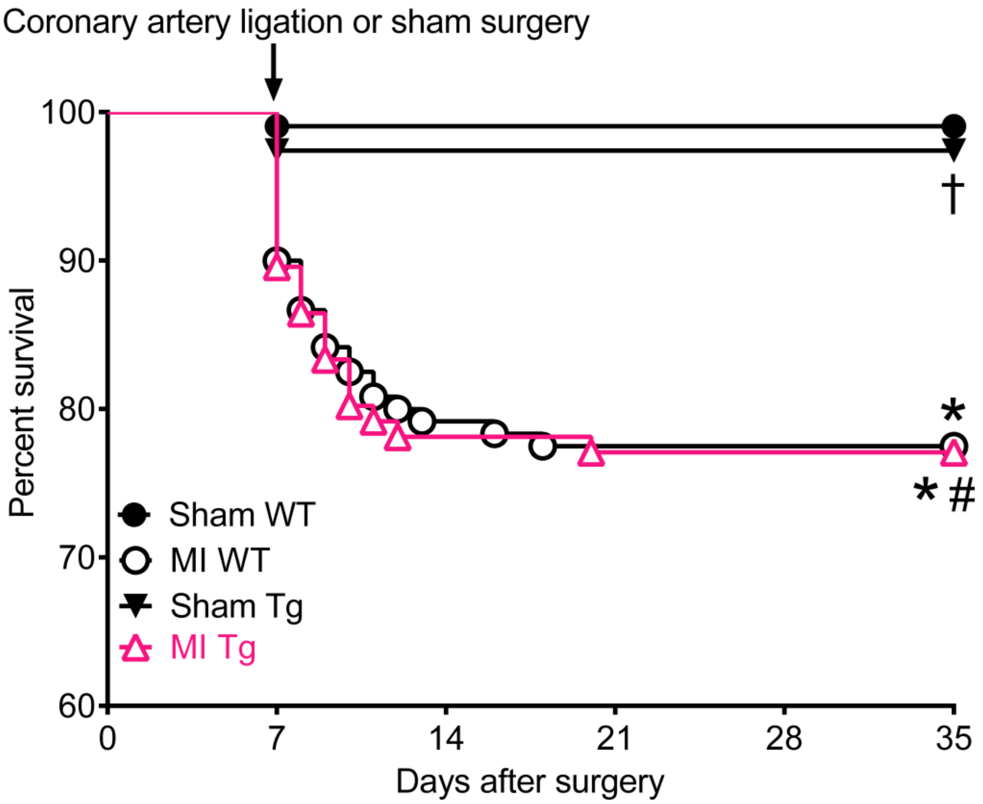

Figure S3 Survival curves for sham-operated and infarcted C57BL/6 wild-type and transgenic mice with cardiomyocyte-specific overexpression of GTP cyclohydrolase 1 (GCH1). Figure legend: sham WT, wild-type mice receiving sham surgery; MI WT, wild-type mice undergoing myocardial infarction; sham Tg, transgenic GCH1 mice receiving sham surgery; MI Tg, transgenic GCH1 mice undergoing myocardial infarction. *P<0.05 versus sham WT group; †P<0.05 versus MI WT group; #P<0.05 versus sham Tg group (n=77-120 mice/group).

Discussion

Potential mechanisms underlying reduced cardiac remodeling after myocardial infarction by GCH1 overexpression

Based on previous studies and our current findings, the possible signaling pathways linking GCH1 to cardiac remodeling and dysfunction after MI are summarized in Figure S4. GCH1 plays a predominant role in cardiac BH_4, a key co-factor for NOS to produce the cardioprotective mediator, NO[21,22]. However, GCH1 proteins are degraded in post-infarction remodeled hearts (Figure 1). Due to decreases in GCH1 and BH_4, three isoforms of NOS: nNOS, iNOS, and eNOS, become monomeric (Figure 8), leading to the switch of the NOS proteins into superoxide ($O_2^{\bullet-}$)-generating enzymes[14,16]. Superoxide is able to react with NO to form a stronger oxidant, peroxynitrite ($ONOO^-$)[23]. $ONOO^-$ as well as $O_2^{\bullet-}$ oxidizes BH_4 to form the catalytically incompetent dihydrobiopterin, leading to further decrease in BH_4 bioavailability[24]. In the meantime, cardiac p38 MAPK is phosphorylated following MI (Figure 4). Genetic and pharmacologic studies reveal that p-p38 MAPK is involved in reduction of SERCA2a and RyR2[25,26]. An increase in p-p38 MAPK results in decreases in RyR2 and SERCA2a proteins (Figure 7). Increases in $O_2^{\bullet-}$ and $ONOO^-$ and decreases in SR Ca^{2+} handling proteins and NO bioavailability together cause interstitial fibrosis in the RZ of myocardium (Figure 3) and cardiac remodeling (Figure 1)[27-29]. nNOS is localized in the SR and plays a regulatory actions on the function of SR Ca^{2+} handling proteins[30]. Dysregulated nNOS and decreased RyR2 and SERCA2a together cause reduction in SR Ca^{2+} release via RyR2 and re-uptake of Ca^{2+} into the SR by SERCA2a (Figure 7)[31]. Thus, myocardial contraction and relaxation are depressed in remodeled myocardium after MI (Figure 5). Cardiomyocyte-specific overexpression of GCH1 increases cardiac GCH1 proteins (Figure 8) and reduces p-p38 MAPK (Figure 4), thereby elevating BH_4 bioavailability, dimeric nNOS (Figures 8 and 9), and SR Ca^{2+} handling proteins (Figure 7). These beneficial changes attenuate cardiac remodeling and dysfunction after MI (Figures 2 and 3).

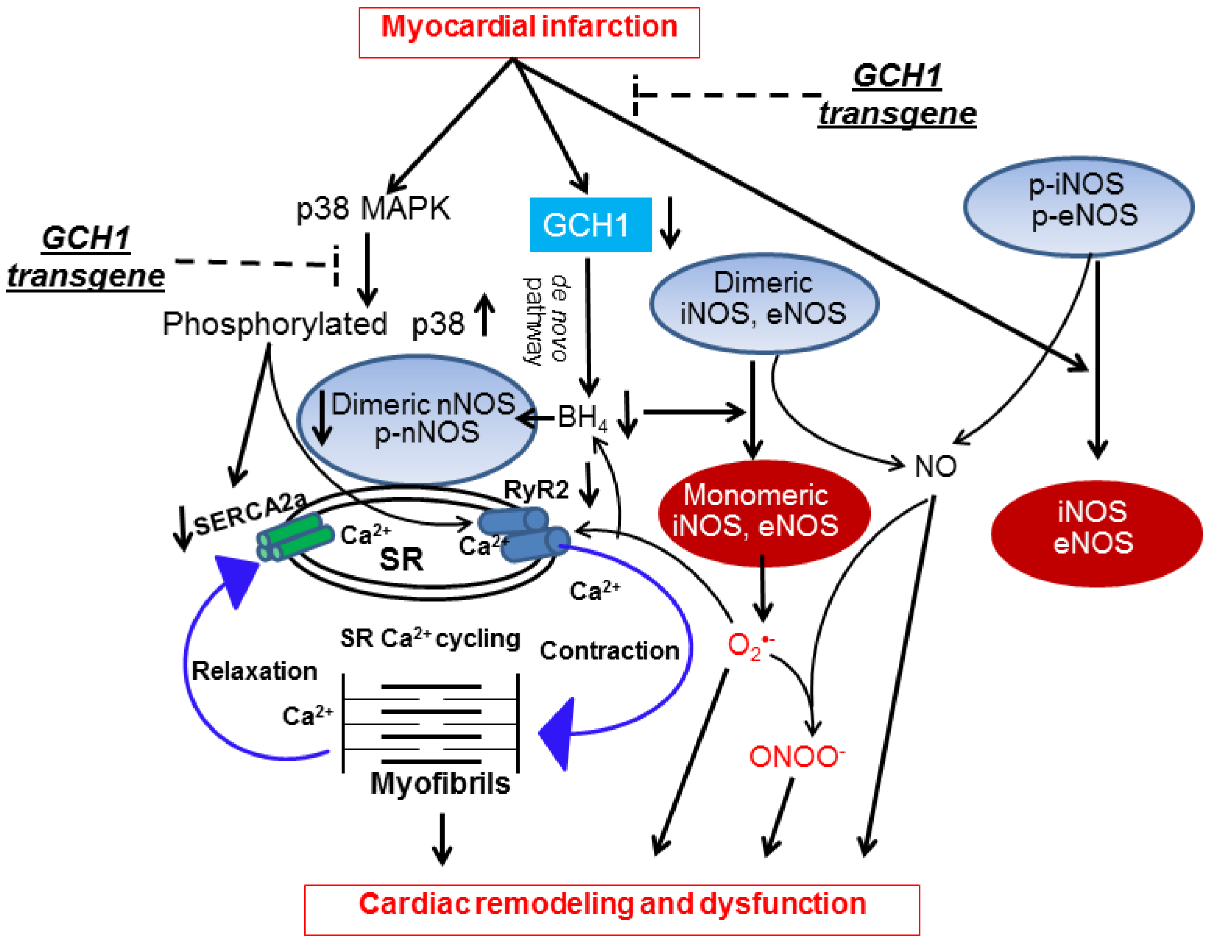

Figure S4 Proposed mechanisms responsible for modulation of postinfarct cardiac remodeling and function by GTP cyclohydrolase 1 (GCH1). GCH1 is the first and rate-limiting enzyme in *de novo* biosynthesis of tetrahydrobiopterin (BH_4). However, GCH1 proteins are decreased in postinfarct remodeled myocardium, starting the early phase of cardiac remodeling. Due to decreases in GCH1 and/or BH_4, the dimers of neuronal nitric oxide synthase (nNOS), inducible NOS (iNOS), and endothelial nitric oxide synthase (eNOS) are decreased, leading to the switch of the nitric oxide synthase proteins into superoxide ($O_2^{\bullet-}$)-generating enzymes. Myocardial infarction results in decreases in phosporylated nNOS (p-nNOS), phosphorylated iNOS (p-iNOS), and phosphorylated eNOS (p-eNOS), thereby causing the reduction of NO. In addition, decreases in GCH1 proteins result in decreases in ryanodine receptors (RyR2) and sarcoplasmic reticulum (SR) Ca^{2+} ATPase (SERCA2a) proteins. nNOS is localized in the SR

and plays a redulatory actions on the function of SR Ca^{2+} handling proteins. Dysfunctional nNOS and decreased RyR2 and SERCA2a together cause reduction in SR Ca^{2+} release via RyR2 and re-uptake of Ca^{2+} into the SR by SERCA2a. Thus, myocardial contraction and relaxation are depressed in remodeled myocardium after myocardial infarction. Cardiomyocyte-specific overexpression of GCH1 elevates cardiac GCH1 and abrogates the detrimental effects of cardiac remodeling on myocardium. Solid arrows indicate direct effects, and dashed arrows indicate the inhibitory effect of transgenic overexpression of GCH1. Blue arrows represent the physiological role of SR Ca^{2+} cycling.

References

1 Ge, Z.D. *et al.* Cardiac-specific overexpression of GTP cyclohydrolase 1 restores ischaemic preconditioning during hyperglycaemia. *Cardiovasc Res* **91**, 340-349 (2011).

2 Ge, Z.D. *et al.* Cl-IB-MECA [2-chloro-N^6-(3-iodobenzyl)adenosine-5'-N-methylcarboxamide] reduces ischemia/reperfusion injury in mice by activating the A_3 adenosine receptor. *J Pharmacol Exp Ther* **319**, 1200-1210 (2006).

3 Ge, Z.D., van der Hoeven, D., Maas, J.E., Wan, T.C. & Auchampach, J.A. A_3 adenosine receptor activation during reperfusion reduces infarct size through actions on bone marrow-derived cells. *J Mol Cell Cardiol* **49**, 280-286 (2010).

4 Baumgardt, S.L. *et al.* Chronic co-administration of sepiapterin and l-citrulline ameliorates diabetic cardiomyopathy and myocardial ischemia/reperfusion injury in obese type 2 diabetic mice. *Circ Heart Fail* **9**, e002424 (2016).

5 Ge, Z.D. *et al.* Isoflurane postconditioning protects against reperfusion injury by preventing mitochondrial permeability transition by an endothelial nitric oxide synthase-dependent mechanism. *Anesthesiology* **112**, 73-85 (2010).

6 Qiao, S. *et al.* MicroRNA-21 mediates isoflurane-induced cardioprotection against ischemia-reperfusion injury via Akt/nitric oxide synthase/mitochondrial permeability transition pore pathway. *Anesthesiology* **123**, 786-798 (2015).

7 Wu, H.E. *et al.* Cardiomyocyte GTP cyclohydrolase 1 protects the heart against diabetic cardiomyopathy. *Sci Rep* **6**, 27825 (2016).

8 Liu, Y. *et al.* Inhibition of PKCβ2 overexpression ameliorates myocardial ischaemia/reperfusion injury in diabetic rats via restoring caveolin-3/Akt signaling. *Clin Sci* **129**, 331-344 (2015).

9 DeWitt, E.S. *et al.* Effects of commonly used inotropes on myocardial function and oxygen consumption under constant ventricular loading conditions. *J Appl Physiol* **121**, 7-14 (2016).

10 Leucker, T.M. *et al.* Impairment of endothelial-myocardial interaction increases the susceptibility of cardiomyocytes to ischemia/reperfusion injury. *PloS ONE* **8**, e70088 (2013).

11 Maczewski, M. & Mackiewicz, U. Effect of metoprolol and ivabradine on left ventricular remodelling and Ca2+ handling in the post-infarction rat heart. *Cardiovasc Res* **79**, 42-51 (2008).

12 Sadredini, M. *et al.* β-Adrenoceptor stimulation reveals Ca^{2+} waves and sarcoplasmic reticulum Ca^{2+} depletion in left ventricular cardiomyocytes from post-infarction rats with and without heart failure. *PloS ONE* **11**, e0153887 (2016).

13 Makarewich, C.A. *et al.* Transient receptor potential channels contribute to pathological structural and functional remodeling after myocardial infarction. *Circ Res* **115**, 567-580 (2014).

14 Vladic, N. *et al.* Decreased tetrahydrobiopterin and disrupted association of Hsp90 with eNOS by hyperglycemia impair myocardial ischemic preconditioning. *Am J Physiol Heart Circ Physiol* **301**, H2130-2139 (2011).

15 Bendall, J.K. *et al.* Stoichiometric relationships between endothelial tetrahydrobiopterin, endothelial NO synthase (eNOS) activity, and eNOS coupling in vivo: insights from transgenic mice with endothelial-targeted GTP cyclohydrolase 1 and eNOS overexpression. *Circ Res* **97**, 864-871 (2005).

16 Bauersachs, J. & Schafer, A. Tetrahydrobiopterin and eNOS dimer/monomer ratio--a clue to eNOS uncoupling in diabetes? *Cardiovasc Res* **65**, 768-769 (2005).

17 Sethumadhavan, S. *et al.* Increasing tetrahydrobiopterin in cardiomyocytes adversely affects cardiac redox state and mitochondrial function independently of changes in NO production. *Free Radic Biol Med* **93**, 1-11 (2016).

18 Saeedi Saravi, S.S., Arefidoust, A., Yaftian, R., Saeedi Saravi, S.S. & Dehpour, A.R. 17α-ethinyl estradiol attenuates depressive-like behavior through GABAA receptor activation/nitrergic pathway blockade in ovariectomized mice. *Psychopharmacology* **233**, 1467-1485 (2016).

19 Prahash, A.J., Gupta, S. & Anand, I.S. Myocyte response to β-adrenergic stimulation is preserved in the noninfarcted myocardium of globally dysfunctional rat hearts after myocardial infarction. *Circulation* **102**, 1840-1846 (2000).

20 Takahashi, T. *et al.* Angiotensin receptor blockade improves myocardial beta-adrenergic receptor signaling in postinfarction left ventricular remodeling: a possible link between beta-adrenergic receptor kinase-1 and protein kinase C epsilon isoform. *J Am Coll Cardiol* **43**, 125-132 (2004).

21 Alkaitis, M.S. & Crabtree, M.J. Recoupling the cardiac nitric oxide synthases: tetrahydrobiopterin synthesis and recycling. *Curr Heart Fail Rep* **9**, 200-210 (2012).

22 Bendall, J.K., Douglas, G., McNeill, E., Channon, K.M. & Crabtree, M.J. Tetrahydrobiopterin in cardiovascular health and disease. *Antioxid Redox Signal* **20**, 3040-3077 (2014).

23 Carballal, S., Bartesaghi, S. & Radi, R. Kinetic and mechanistic considerations to assess the biological fate of peroxynitrite. *Biochim Biophys Acta* **1840**, 768-780 (2014).

24 Landmesser, U. *et al.* Oxidation of tetrahydrobiopterin leads to uncoupling of endothelial cell nitric oxide synthase in hypertension. *J Clin Invest* **111**, 1201-1209 (2003).

25 Arai, M. *et al.* Mechanism of doxorubicin-induced inhibition of sarcoplasmic reticulum Ca^{2+}-ATPase gene transcription. *Circ Res* **86**, 8-14 (2000).

26 Auger-Messier, M. *et al.* Unrestrained p38 MAPK activation in Dusp1/4 double-null mice induces cardiomyopathy. *Circ Res* **112**, 48-56 (2013).

27 Takimoto, E. & Kass, D.A. Role of oxidative stress in cardiac hypertrophy and remodeling. *Hypertension* **49**, 241-248 (2007).

28 Kohr, M.J., Roof, S.R., Zweier, J.L. & Ziolo, M.T. Modulation of myocardial contraction by peroxynitrite. *Front Physiol* **3**, 468 (2012).

29 Sedej, S. *et al.* Subclinical abnormalities in sarcoplasmic reticulum Ca^{2+} release promote eccentric myocardial remodeling and pump failure death in response to pressure overload. *J Am Coll Cardiol* **63**, 1569-1579 (2014).

30 Zhang, Y.H., Jin, C.Z., Jang, J.H. & Wang, Y. Molecular mechanisms of neuronal nitric oxide synthase in cardiac function and pathophysiology. *J Physiol* **592**, 3189-3200 (2014).

31 Loyer, X. *et al.* Cardiomyocyte overexpression of neuronal nitric oxide synthase delays transition toward heart failure in response to pressure overload by preserving calcium cycling. *Circulation* **117**, 3187-3198 (2008).
